# Efficacy and cost of acoustic-informed and wind speed-only turbine curtailment to reduce bat fatalities at a wind energy facility in Wisconsin

**DOI:** 10.1371/journal.pone.0266500

**Published:** 2022-04-08

**Authors:** Paul A. Rabie, Brandi Welch-Acosta, Kristen Nasman, Susan Schumacher, Steve Schueller, Jeffery Gruver

**Affiliations:** 1 Western EcoSystems Technology, Inc., Laramie, Wyoming, United States of America; 2 Western EcoSystems Technology, Inc., Cheyenne, Wyoming, United States of America; 3 Western EcoSystems Technology, Inc., Fort Collins, Colorado, United States of America; 4 We Energies, Milwaukee, Wisconsin, United States of America; 5 Rocky Mountain Bat Conservancy, Laramie, Wyoming, United States of America; Centro de Investigacion Cientifica y de Educacion Superior de Ensenada, MEXICO

## Abstract

Current research estimates hundreds of thousands of turbine-related bat fatalities in North America annually. In an effort to reduce impacts of wind energy production on bat populations, many facilities implement operational curtailment strategies that limit turbine blade rotation during conditions when nighttime wind speeds are low. Incorporating real-time bat activity data into wind speed-only curtailment (WOC) strategies may increase operational flexibility by allowing turbines to operate normally when bats are not present near turbines. We evaluated costs and benefits of implementing the Turbine Integrated Mortality Reduction (TIMR) system, an approach that informs a curtailment-triggering algorithm based on wind speed and real-time bat acoustic data, compared to a WOC strategy in which turbines were curtailed below 4.5 meters per second (m/s) at a wind energy facility in Fond Du Lac County, Wisconsin. TIMR is a proprietary system and we had no access to the acoustic data or bat call analysis software. Operational parameters for the TIMR system were set to allow curtailment at all wind speeds below 8.0 m/s during the study period when bats were acoustically detected. Overall, the TIMR system reduced fatalities by 75% compared to control turbines, while the WOC strategy reduced fatalities by 47%. An earlier analysis of the same TIMR data neglected to account for carcasses occurring outside the plot boundary and estimated an 84.5% fatality reduction due to the TIMR system. Over the study period, bat activity led to curtailment of TIMR turbines during 39.4% of nighttime hours compared to 31.0% of nighttime hours for WOC turbines, and revenue losses were approximately 280% as great for TIMR turbines as for turbines operated under the WOC strategy. The large cost difference between WOC and TIMR was driven by the 4.5 m/s versus 8.0 m/s wind speed thresholds for curtailment, but our study site has a relatively low average wind speed, which may also have contributed; other wind operators considering the TIMR system will need to consider their ability to absorb production losses in relation to their need to reduce bat fatality rates.

## Introduction

Bat populations in North America face numerous threats, among which white-nose syndrome and wind turbine collisions have become the leading causes of mortality since 2000 [1]. This coincides with the increase of utility-scale wind turbine installations over the past 20 years [2]. Installed land-based capacity of wind power in the US increased from about 2.5 gigawatts (GW) to more than 105 GW (105,583 megawatts [MW]) between 1999 and 2019 [2]. By 2050, land-based wind power capacity is projected to reach over 400 GW through the installation of larger, more efficient turbines and an increasing number of wind energy facilities [3]. Published estimates range from hundreds of thousands of turbine-related bat fatalities in North America each year [4, 5], to more than a million fatalities [6], raising concern over the long-term impacts that bat populations may experience given the increasing magnitude of wind energy development [7, 8].

In the US, increased attention to turbine-related bat fatalities followed the 2004 discovery of high levels of bat collisions at wind energy facilities in West Virginia and Pennsylvania [9]. Available studies consistently indicate bat fatalities at wind turbines are greatest during nights with wind speeds less than 6.0 m/s. The operational “cut-in speed”, the speed at which turbines begin to produce energy [10], of most modern wind turbines is 3.0–4.0 m/s; however, free-spinning blades below cut-in speed can also pose a collision hazard [11]. Minimizing blade rotation below operational cut-in speed by “feathering” (positioning blades parallel to the wind to reduce aerodynamic lift and slow rotation to a near halt [less than 1 rotation per minute]), and increasing cut-in speed can reduce collision fatalities [11].

Increasing cut-in speed results in a decrease of electric energy produced and uploaded to the grid [10]. We refer to operational curtailment based on wind speed as “wind speed-only curtailment” (WOC) because the primary determinant of when feathering turbine blades occurs is wind speed. Arnett et al. [11] summarized the findings of 10 WOC studies and reported that most observed a 50% or greater reduction of bat fatalities when operational cut-in speeds of 3.0–4.0 m/s were raised by 1.5 m/s. Curtailment, by definition, results in some reduction in energy generation and loss of revenue. Few studies have disclosed operational costs associated with curtailment, but those that have report estimated annual power production reduction of at least 1% [11].

Stakeholders and energy developers have expressed interest in identifying more efficient curtailment strategies that will not only reduce impacts of wind energy production on wildlife, but also reduce total curtailment time, thereby minimizing losses in generation and revenue [12, 13]. Technological advances in acoustic detection of bat activity near turbines [13–15] may provide a means for incorporating real-time bat activity data into curtailment strategies, (but see Voigt et al. [16] for a discussion of limitations to acoustic monitoring). If successful, such “informed curtailment” could allow turbines to operate fully during times that a traditional WOC strategy would not (i.e., when bats are not present), increasing operational flexibility [12].

A 2015 study at the Blue Sky Green Field Wind Energy Facility (BSGF) tested efficacy (i.e., mortality reduction) of the Turbine Integrated Mortality Reduction^SM^ (TIMR; Normandeau Associates, Bedford, New Hampshire) system [12]. The TIMR system is a proprietary, commercial informed curtailment strategy developed by Normandeau Associates and the Electric Power Research Institute. This system uses a risk assessment algorithm derived from real-time bat acoustic detection data and site-specific wind speed data to control turbine operations. Reduction in mortality of all bats was reported to be 84.5%, with an estimated 3.2% reduction in annual revenue compared to turbines that were not feathered [12]. But a better understanding of benefits and costs of informed curtailment requires simultaneous evaluation of informed curtailment and a WOC treatment in terms of efficacy and cost. We report additional data collected concurrent to the study at the BSGF [12] to evaluate efficacy and costs of the TIMR system along with a traditional WOC strategy with a raised cut-in speed of 4.5 m/s.

In addition to adding the evaluation of WOC with TIMR, we re-analyzed the TIMR data with explicit attention to the spatial distribution of carcasses within plots. There is a growing awareness that turbine curtailment may alter the spatial distribution of bat carcasses beneath wind turbines because the distance a bat travels following collision with a turbine blade may depend on wind speed at the time of collision [17]. Wind speed during collision events is likely to be higher (on average) at curtailed turbines than at control turbines, suggesting that the carcass distribution at curtailed turbines may be concentrated further from turbine bases, compared to control turbines. In cases where plots are relatively small (square plots with a 80-m side length in this study and the study by Hayes et al. [12]), estimated efficacy of a curtailment treatment may be exaggerated if the proportion of carcasses falling outside of search areas is greater for the curtailment treatment than for the control treatment.

Our objectives were to 1) estimate reduction in bat fatalities attributed to each curtailment strategy relative to control turbines operated with no curtailment and no feathering, 2) estimate the economic loss in power generation for each curtailment strategy, and 3) compare cost (relative economic loss in power generation and revenue) and benefit (fatality reduction) between each curtailment strategy.

## Methods

The work reported here was completed under the following permit: Wisconsin Department of Natural Resources Endangered and Threatened Species Permit #934 issued to JG.

### Study area

The BSGF is a 145-MW wind power production site operated by We Energies in northeastern Fond du Lac County, Wisconsin (43°52′46″N, 88°16′15″W). The facility became operational in 2008 and consists of 88 monopole Vestas V82 turbines (hub height of 80 m above ground level, rotor diameter of 82 m) located on approximately 42 square kilometers. The landscape is characterized by flat to rolling topography, dominated by cultivated agriculture and dairy farming, and interspersed with small woodland patches. The most abundant crop was corn (*Zea mays*), though soybean (*Glycine max*), alfalfa (*Medicago sativa*), and winter wheat (*Triticum aestivum*) fields were also present. Elevation ranges from approximately 240−335 m above mean sea level and the county has a humid continental climate (mean annual temperature about 7.5 degrees Celsius, with total precipitation of about 76.2 centimeters). The average annual wind speed on site was 6.3 m/s, with lowest speeds observed during July and August.

In a post-construction fatality study conducted at the BSGF in 2008 − 2009, carcasses from 5 species of bats were discovered: little brown bat (*Myotis lucifugus*), big brown bat (*Eptesicus fuscus*), silver-haired bat (*Lasionycteris noctivagans*), hoary bat (*Lasiurus cinereus*), and eastern red bat (*Lasiurus borealis*) [18]. Drake et al. [19] reported tri-colored bat (*Perimyotis subflavus*) and northern long-eared bat (*Myotis septentrionalis*) occurrences in Wisconsin at the Neda Mine, about 56 kilometers southwest of the BSGF, where they were recorded hibernating along with other species, but these 2 species were not observed during the 2008–2009 study at the BSGF [18].

### Study design

Among the 88 turbines at the BSGF, 30 were selected for inclusion in the current (2015) study using a systematic design with a random start. Some turbines were not made available for selection because landowners declined to provide access. Selected turbines were randomly assigned to 1 of 3 treatment groups, constrained to 10 turbines per group. Although it is preferable to rotate treatments through turbines to ensure that spatial gradients in mortality do not influence the study, logistical constraints imposed by the TIMR treatment (i.e., labor-intensive equipment installation) precluded that approach and we relied on random assignment of treatments to turbines to minimize the effects of any inherent underlying variation among turbines.

The first group served as a control. Turbines were allowed to operate normally with a cut-in speed of 3.5 m/s and the ability to free spin (i.e., blades were not feathered) when power was not being generated. Another group operated under traditional WOC constraints, with turbine blades feathered below a cut-in speed of 4.5 m/s, 1.0 m/s above the manufacturer’s rating of 3.5 m/s. Curtailment of the third group of turbines was controlled by the TIMR system, as described below. Each curtailment strategy was applied from 1800–0600 hours from July 15 –August 31, and from 1800–0700 hours from September 1 –September 30, the expected highest bat activity periods. Start and stop times were set to ensure that treatments were in place from sunset until sunrise, and the BSGF turbine control system was constrained to making changes on an hourly basis.

### Turbine Integrated Mortality Reduction system

The TIMR system curtailed turbines based on real-time acoustic bat activity data and wind speeds. A proprietary bat detection and monitoring system (ReBAT®; Normandeau Associates, Bedford, New Hampshire) informed TIMR. ReBAT used full-spectrum ultrasonic detectors and proprietary noise filters to process acoustic data to determine when bat calls were detected. Information about bat detections was transmitted in real time to the TIMR system. ReBAT units were mounted on 4 turbine nacelles under Normandeau Associates’ guidance, and positioned to sample airspace in the directions bats were predicted to fly based on the wind regimes observed on site, which included the northern, northwestern, southwestern, and southeastern parts of the wind facility [12, 20]. Wind speed data from the anemometer associated with 1 of these turbines was transmitted to the TIMR system. Turbines with ReBAT units were selected to maximize spatial coverage of the wind facility, and not all of the turbines with ReBAT units were TIMR treatment turbines. Maximizing spatial coverage over the wind facility rather than concentrating the ReBAT units on the TIMR treatment turbines means that the TIMR treatment may not have been as effective as it could have been; however, the general deployment pattern (acoustic detectors and anemometers on a subset of turbines) was consistent with the general deployment scheme for the TIMR curtailment system [20]. Data were monitored in fixed, 10-minute increments, and all TIMR treatment turbines were curtailed when 10-minute average wind speeds were less than 8.0 m/s and at least 1 bat call had been detected at any of the ReBAT units within the previous 30 minutes. Under these conditions, the TIMR system would cease turbine operations and feather blades to reduce collisions. Blades remained feathered until the risk of bat fatalities was low (i.e., no bat calls within 30 minutes or average wind speed was greater than 8.0 m/s). Under low risk conditions, when no bat calls had been recorded for 30 minutes, turbines operated normally with blades feathered below the 3.5 m/s cut-in speed.

The choice of 8.0 m/s as a wind speed threshold for the TIMR treatment is higher than typical turbine curtailment thresholds. A high wind speed threshold was used for this early test of TIMR because the test was in part proof-of-concept. The 8.0 m/s threshold also means that we do not have a comparison between 2 treatments with identical wind speed thresholds, but the study design enables us to estimate efficacy (i.e., fatality reduction) and cost of each treatment, and evaluate the cost-benefit tradeoff for the anticipated enhanced efficacy of the TIMR treatment relative to the WOC treatment.

The proprietary nature of the TIMR/ReBAT system means that we did not have access to the underlying bat acoustic data, the bat call filtering algorithms, detector calibration data, or data from the single anemometer that informed the TIMR system (but some of those data are summarized elsewhere, see Electrical Power Research Institute [20]). Although bat activity patterns and the performance of the call filtering algorithms are interesting in their own right, the analysis in the present study is limited to efficacy and cost of the curtailment treatments.

### Bat fatality surveys

Fatality estimation consisted of 4 primary components: 1) standardized carcass surveys at study turbines, 2) searcher efficiency (SEEF) trials to estimate the percentage of carcasses found by searchers, 3) carcass persistence trials to estimate the probability that a carcass remained in the field for possible detection, and 4) data analysis to determine adjusted fatality estimates for the study period.

#### Bat fatality surveys and estimation

Carcass searches were conducted daily at each study turbine from July 15 − September 30, 2015. Occasionally, weather or turbine maintenance precluded a search on a particular day. Previous fatality monitoring at the BSGF indicated that 89% of carcasses fell within 40 m (131 ft) of turbine towers ([18], but see discussion); therefore, square survey plots (80 x 80 m) centered on each study turbine were established. Vegetation was cleared by periodic mowing and herbicide application to facilitate search efforts. Within each survey plot, transects spaced 5.0 m apart were walked by trained searchers at a pace of approximately 45–60 m per minute. Searchers scanned 3.0 m on both sides of each transect for bat carcasses, allowing for a 1.0-m overlap between transects.

All carcasses located within search plots were recorded using standardized data sheets, and data included species, sex and age (when possible), date and time collected, global positioning system location, condition (e.g., intact, scavenged), distance from the turbine, and approximate time since death. Each carcass was photographed as found and plotted on a detailed map of the corresponding survey plot. All carcasses were given a unique identification code, collected, and stored in a freezer at the BSGF. Injured bats observed during surveys were recorded and treated as fatalities for the purpose of analysis. Carcasses found outside of a scheduled search within a survey plot were treated as a documented fatality under the assumption that the carcass would have been found during the next scheduled search. Fatalities observed outside of delineated search plots were recorded, but were not included in the fatality estimates.

#### Searcher efficiency trials

The SEEF trials were used to estimate the proportion of carcasses found by searchers and to correct for detection bias in fatality estimates. All SEEF trials were conducted within standardized survey plots on 12 days throughout the study period. One to 4 bat carcasses collected previously at the BSGF were placed at pre-determined locations within the survey area prior to the standardized carcass searches. Carcasses were placed at random directions and distances from wind turbines within search plots to ensure a representative sample of search substrates. Searchers performing standardized carcass surveys did not know when SEEF trials were being conducted or in which survey plots carcasses were placed. Each placed carcass was discreetly marked so that it could be easily identified as a SEEF carcass when found. Immediately after each trial, the number of SEEF carcasses available for detection and the number recovered by searchers were determined; carcasses that had been scavenged or were otherwise unavailable to searchers were excluded from SEEF estimates. Throughout the study period, a total of 42 SEEF carcasses were placed within search plots.

#### Carcass persistence trials

Carcass persistence trials were also conducted in the same plots as standardized surveys to estimate the probability that carcasses would be removed by scavengers or other means (e.g., being mowed over during vegetation management practices, or moved by cattle [*Bos taurus*] or agricultural machinery), and be unavailable to searchers. Trial carcasses were placed randomly within survey plots and monitored for 30 days. Placed carcasses were checked every day for the first 4 days, then again on days 7, 10, 14, 20, and 30, or until removed from the survey plot. The survey schedule varied slightly when weather or coordination with the other survey work prevented carcass persistence trial checks. In total, 56 bat carcasses were placed throughout the study period to estimate persistence probability during the study.

### Statistical analysis

#### Fatality rate estimation

We used the GenEst mortality estimator [21–23] to obtain fatality estimates for each group (control, WOC, TIMR) of study turbines. Fatality estimates were based on all bat carcasses found on search plots. The GenEst mortality estimator adjusts carcass counts based on searcher efficiency, search interval, carcass persistence, and the fraction of carcasses expected to fall beyond the bounds of the search plot. Carcass persistence (i.e., the probability that a bat carcass would persist and be available to searchers at the next search) was modeled using survival regression methods [21]. Four persistence time distributions (log-normal, log-logistic, Weibull, and exponential) were fitted to trial data, and sample-size adjusted (corrected) Akaike’s Information Criterion [24] was used to select the best-fitting model. Carcass persistence models were fitted without covariates. Searcher efficiency was modeled using single-search bias trials (i.e., searchers had exactly 1 opportunity to discover a trial carcass) and no covariates. When GenEst is used with single-search trial data, it is necessary to supply the estimator with a value for the detection reduction factor (sometimes called *k*, which takes values between 0 and 1) [23]. The parameter, *k*, accounts for reduced detectability of carcasses through time; for a carcass that is not detected the first time it is available to searchers, the SEEF, *p*, will be reduced to *pk* on the second search, *pk*^*2*^ on the third search, and so forth. At the time of writing, GenEst does not estimate the adjustment for carcasses falling beyond the boundaries of searched plots, but rather accepts an input “area correction” value that indicates the fraction of carcasses expected in search areas.

We modeled the spatial distribution of carcasses around turbines by fitting gamma, Gompertz, Rayleigh, truncated normal, and Weibull distributions to the carcasses’ distance from turbine data, and used corrected Akaike’s Information Criterion to select the best fitting distribution for each of our 3 treatments. Because our search areas were squares with 80-m length sides, our carcass distance data were right-truncated at a distance of 40×√2 = 56.6 m at the corners of plots. Proportion of area searched in each 1.0-m annulus around turbines was 1.0 between 0 and 40 m, but decreased to near 0 from 40 m to the corner of the plot. To account for variable search effort within our search areas, carcass distance distributions were fitted with a truncated weighted likelihood (TWL) approach [25]. The TWL method uses maximum likelihood to fit a density distribution to right-truncated data, but each observation is up-weighted in the likelihood to account for its inclusion probability in the data set (i.e., the proportion of area searched at its distance). Having obtained a carcass density distribution, the area correction value was calculated by multiplying the fraction of carcasses predicted in each 1.0-m annulus around the turbine by the fraction of area searched in that annulus and summing over the search plot [25]. We estimated 90% confidence intervals (CI) by drawing 1,000 bootstrap samples of carcass distances for each of the 3 treatments, refitting the carcass distribution model using the same distribution indicated for the point estimate, and recalculating the area correction value. The use of a resampling procedure rather than a parametric bootstrap accounts for uncertainty in the carcass sample.

Estimated reductions in fatality were considered different from one another if their 90% CIs did not overlap. Fatality estimates produced by GenEst include robust CIs, but not an estimation of the variance, precluding use of a parametric statistical test for differences among treatments.

#### Comparison of turbine operation and revenue

We examined variation in turbine operation and estimated the effects of each curtailment strategy on power generation and revenue compared to control turbines. For each curtailment strategy, we compared average wind speed (m/s) across the study period during potential curtailment hours (1800–0600 hours from July 15 –August 31, and 1800–0700 hours from September 1 − 30), average percent of time turbines were curtailed, estimated loss in power generation due to curtailment (MW), and estimated loss in revenue due to curtailment. Lost power generation due to curtailment was estimated as the difference in power generated by each treatment group. This approach implicitly assumes that in the absence of experimental treatments, power generation would have been similar between groups of turbines. Revenue losses were calculated assuming a gross potential market revenue value of $40/MW [20], an appropriate estimate for the timeframe of the study [12].

## Results

### Bat fatality surveys

Across all 30 survey plots, 2,268 of 2,310 planned searches (98%) were completed and 296 bats representing 6 species were found (Table 1). Similar to other curtailment studies, the most common bat fatalities (69.3%, n = 205) were migratory tree bats (i.e., hoary bat, silver-haired bat, eastern red bat). The little brown bat and big brown bat were also relatively common. Only 2 tri-colored bats and no northern long-eared bats were found, despite their known occurrence near the BSGF. Bat fatalities occurred throughout the study period, with peak fatalities occurring in August.

**Table 1:** Observed bat fatalities included in analysis for 30 study turbines at the Blue Sky Green Field Wind Energy Facility, Fond du Lac County, Wisconsin, from July 15, 2015 − September 30, 2015.

Species	Control plots^a^		WOC plots^a^		TIMR plots^a^		Total^a^	
	Total	Percent	Total	Percent	Total	Percent	Total	Percent
**Big brown bat**	27	14.4	8	10.3	7	22.6	42	14.2
**Eastern red bat**	37	19.8	19	24.4	6	19.4	62	20.9
**Hoary bat**	48	25.7	18	23.1	11	35.5	77	26.0
**Little brown bat**	29	15.5	15	19.2	3	9.7	47	15.9
**Silver-haired bat**	45	24.1	17	21.8	4	12.9	66	22.3
**Tri-colored bat**	1	0.5	1	1.3	0	0	2	0.7
**Totals**	**187**	**100**	**78**	**100**	**31**	**100**	**296**	**100**

^a^Data were collected at plots for 10 control turbines, 10 wind speed-only curtailed (WOC) turbines with a cut-in speed of 4.5 meters per second, and 10 Turbine Integrated Mortality Reduction (TIMR) system-curtailed turbines.

### Fatality rate estimates

**Fig 1:**
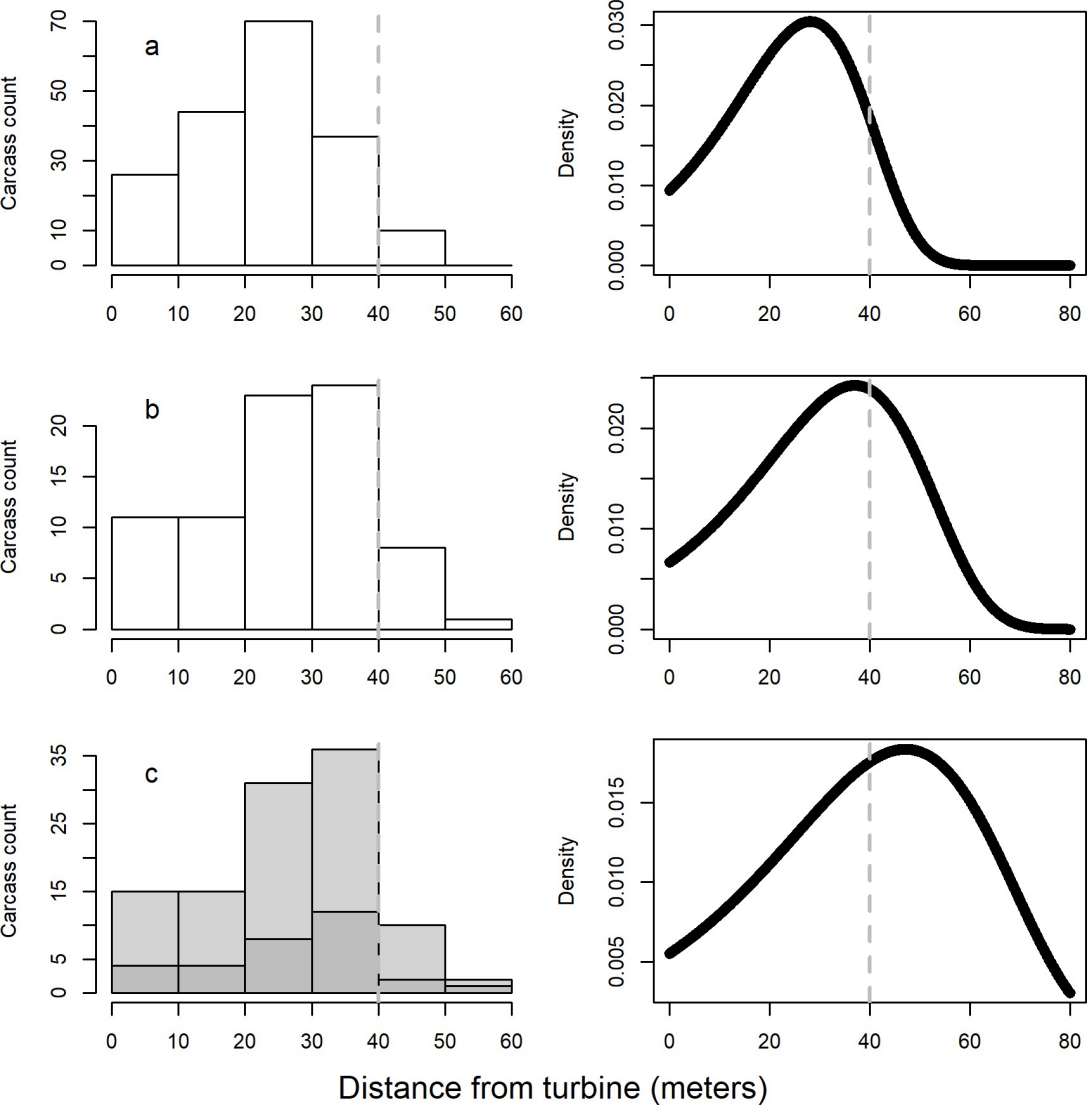
Distances of carcasses from turbine bases, and fitted curves describing the carcass density distribution. Model selection indicated Gompertz density distributions as the best fit in all cases. Grey dashed vertical lines on the plots indicate the 40-m threshold beyond which search effort decreased because only the corners of the square plots were searched beyond 40 m. Data were collected at 10 control turbines (Control; panel a), 10 wind speed-only curtailed (WOC) turbines with a cut-in speed of 4.5 m/s (panel b), and 10 Turbine Integrated Mortality Reduction (TIMR) system-curtailed turbines (panel c). Panel c indicates locations of the carcasses collected at the TIMR plots in dark grey bars, and the combined WOC + TIMR data used to fit the model in light gray bars, because the TIMR-only data produced an implausible carcass density distribution.

Our total count of bat carcasses included 2 additional hoary bat carcasses from the TIMR-treatment turbines that were not reported in Hayes et al. [12], apparently due to an oversight. The estimated SEEF rate was 60% (90% CI: 47%, 71%) and median carcass persistence time was 8.7 days (90% CI: 5.6 days, 13.3 days) with a lognormal persistence time distribution, resulting in a 94% persistence probability for our daily search regime. Area correction values for control and WOC treatments were 0.92 (90% CI: 0.87, 0.95) and 0.72 (90% CI: 0.37, 0.89), respectively. However, the area correction value for the TIMR treatment was implausibly low (much less than 0.01) because the right-truncated carcass distance data for this treatment did not clearly include the mode of the distribution (Fig 1). The TWL fitting method for carcass density distributions is prone to failure when the search radius is insufficient to capture the mode of the carcass fall distribution [25]. We combined carcass distance data from the TIMR treatment with distance data from the WOC treatment and obtained an area correction value of 0.51 (90% CI: 0.18, 0.87). This value is probably too high, so our estimate of fatality for the TIMR treatment may be artificially low. Fitted distributions and parameters are given in Table 2.

**Table 2:** Fitting details for the carcass density distribution models for the control treatment, wind-only curtailment treatment, Turbine Integrated Mortality Reduction treatment, and for combined wind-only curtailment treatment and Turbine Integrated Mortality Reduction treatment^a^.

Treatment^b^	Number of carcasses	Shape	Scale	Area correction estimate (90% CI)^c^
**Control**	187	0.0728	0.0094	0.92 (0.87, 0.95)
**WOC**	78	0.0589	0.0066	0.72 (0.37, 0.90)
**TIMR**	31	0.038	<0.0001	<< 0.001
**TIMR + WOC**	109	0.0441	0.0055	0.52 (0.19, 0.87)

^a^Model selection indicated a Gompertz distribution for all carcass distances in all treatments.

^b^Treatment groups included 10 control turbines (Control), 10 wind speed-only curtailed (WOC) turbines with a cut-in speed of 4.5 meters per second, and 10 Turbine Integrated Mortality Reduction (TIMR) system-curtailed turbines.

^c^CI, confidence interval

Mortality was reduced by 47% at WOC turbines compared to control turbines (90% CI: -1%, 60%, where negative numbers indicate increased mortality and a finding of no difference), and the TIMR strategy reduced fatalities by 75% (90% CI: 13%, 84%) compared to control turbines (Table 3). The TIMR treatment reduced fatality rates by 1.5 (90% CI: -1.1, 4.1) times more than the WOC treatment.

**Table 3:** Observed and estimated bat fatalities for 3 treatment groups at the Blue Sky Green Field Wind Energy Facility, Fond du Lac County, Wisconsin, from July 15 − September 30, 2015^a^.

Treatment^b^	Observed fatalities number/turbine/study period	Adjusted fatalities number/turbine/study period (90% CI)	Adjusted fatalities number/MW/study period (90% CI)^c^
Control	18.70	26.28 (23.54, 30.96)	15.95 (14.28, 18.79)
WOC	7.80	14.12 (10.54, 27.14)	8.57 (6.4, 16.47)
TIMR	3.10	6.62 (4.23, 23.68)	4.02 (2.57, 14.37)

^a^Estimates were made using the GenEst fatality estimator.

^b^Treatment groups included 10 control turbines (Control), 10 wind speed-only curtailed (WOC) turbines with a cut-in speed of 4.5 meters per second, and 10 Turbine Integrated Mortality Reduction (TIMR) system-curtailed turbines.

^c^CI, confidence interval; MW, megawatt.

### Comparison of turbine operation and revenue

Relative to control group turbines, those in the WOC and TIMR groups operated less often when conditions were suitable for power generation. On average, turbines in the WOC treatment were curtailed for 31.0% of the nighttime treatment period, while turbines in the TIMR group were curtailed 39.4% of the nighttime treatment period. Over the 78-day study period (July 15 –September 30), WOC and TIMR turbines generated 5.3% and 14.8% less power than control turbines, respectively (Table 4). Average estimated revenue loss for TIMR turbines was approximately 2.8 times as great as for WOC turbines ($3,597.84/turbine for TIMR compared to $1,291.76/turbine for WOC), when compared to revenue generated by control turbines (Table 5).

**Table 4:** Observed power production for 3 treatment groups at the Blue Sky Green Field Wind Energy Facility, Fond du Lac County, Wisconsin, July 15 − September 30, 2015.

Treatment^a^	Average wind speed (m/s)^b^	Average power generation (MWh/turbine)^c^^,^^d^	Revenue (dollars/turbine)^d^
**Control**	5.8	606.3	$24,253.80
**WOC**	5.7	574.1	$22,962.04
**TIMR**	5.6	516.5	$20,661.96

^a^Values incorporate experimental treatment effects and variation due to turbine maintenance and other factors.

^b^m/s, meters per second.

^c^MWh, megawatt-hours.

^d^Treatment groups included 10 control turbines (Control), 10 wind speed-only curtailed WOC turbines with a cut-in speed of 4.5 m/s, and 10 Turbine Integrated Mortality Reduction TIMR system-curtailed turbines.

**Table 5:** Power loss based on differences in observed power production for 3 treatment groups at the Blue Sky Green Field Wind Energy Facility, Fond du Lac County, Wisconsin, July 15 − September 30, 2015.

Comparison^a^	Power generation difference (MWh/turbine)^b^^,^^c^	Revenue difference (dollars/turbine)^c^
**Control vs. TIMR**	89.8	$3,597.84
**Control vs. WOC**	32.3	$1,291.76
**WOC vs. TIMR**	57.5	$2,300.08

^a^Values include variation from turbine maintenance and other factors.

^b^MWh, megawatt hours.

^c^Treatment groups included 10 control turbines (Control), 10 wind speed-only curtailed WOC turbines with a cut-in speed of 4.5 meters per second, and 10 Turbine Integrated Mortality Reduction TIMR system-curtailed turbines.

## Discussion

To our knowledge, this is the first controlled experiment to present costs and fatality reductions of near real-time informed curtailment (i.e., TIMR) and traditional WOC at a wind energy facility in North America. Hayes et al. [12] estimated fatality reductions due to the TIMR system at this site, but did not adjust fatality estimates for carcasses falling outside of searched areas and did not include the data from the WOC treatment, and so could not compare fatality reductions or production losses between the 2 curtailment strategies. Our results suggest that the TIMR strategy results in fewer bat fatalities than the WOC strategy, but uncertainty in the area correction means that our estimate is a maximum bound on the difference. Our relatively small sample sizes contributed to wide CIs around our estimates.

We anticipated greater revenue losses from the TIMR strategy compared to the WOC strategy because the system was set to curtail operation up to 8.0 m/s (26.2 ft/s), compared to 4.5 m/s for WOC. We found that the TIMR strategy was up to 1.5 times more effective than the WOC strategy at 2.8 times the cost. Relative costs and benefits of the 2 strategies probably do not follow a linear relationship as cut-in speed thresholds change. Were the 2 strategies implemented with the same cut-in speed, we would expect WOC to be at least as effective as TIMR, and TIMR to be at most as costly as WOC because WOC would be guaranteed to curtail turbines whenever TIMR curtailed turbines, but the converse would not be true. Tests are needed across a range of cut-in speeds to develop a complete understanding of the relative costs and benefits of the TIMR system.

Few studies in North America have reported metrics for economic losses associated with WOC. In Alberta, Canada, Baerwald et al. [26] reported total estimated economic losses of $3,000−$4,000 (Canadian dollars) for 15 turbines curtailed 24 hours a day below a raised cut-in speed of 5.5 m/s (relative to a baseline cut-in speed of 4.0 m/s) from August 1 –September 7. If turbines had been curtailed only during nighttime hours when bats are active, economic losses would have been reduced [26]. In Pennsylvania, Arnett et al. [27] reported estimated losses of 3% of study-period production for turbines curtailed nightly to a raised cut-in speed of 5.0 m/s (from a baseline of 3.5 m/s) and 11% of study-period production for turbines with a raised cut in speed of 6.5 m/s from 27 July– 9 October; this was a larger difference in relative cost for a smaller difference in cut-in speed than in the present study, indicating the utility of acoustic-triggered curtailment in the TIMR treatment. The relatively high percentage production loss at the BSGF compared to other North American studies may be the result of the low wind speed regime at the BSGF [20], which leads to more frequent curtailment events at a set cut-in speed.

Studies that have raised cut-in wind speeds at turbines to investigate the resulting reduction in bat fatalities have tested WOC under thresholds ranging from 3.0−6.5 m/s, and reported mean reductions in bat mortality ranging from 34%−82% [6, 11, 26–28]. During the present study, the WOC strategy resulted in a mean reduction in bat fatalities (47%) that is within the range of reductions reported by other studies, and similar to the findings reported for the Anonymous Project 1 in US Fish and Wildlife Region 3 in 2010 (47%), where cut-in speeds were also raised to 4.5 m/s [11]. We are unaware of any publicly available studies that have investigated a curtailment approach that incorporates near real-time bat acoustic data at a North American wind energy facility other than the BSGF [12]. The estimated reduction in fatalities with the TIMR system compared to control turbines (75%) at BSGF was higher than most same-site-same-year comparisons of WOC to control reported to date but lower than the 85% reduction reported by Hayes et al. [12]. Additional studies or longer-term studies will help to determine how representative these results are of TIMR performance.

Some caution is needed in the interpretation of our fatality reduction results (and the fatality reduction results presented in most studies of curtailment to date). Previous studies have not applied treatment-specific adjustments for carcasses occurring outside of survey plots. In the present study, we found that increasing cut-in speed from 3.5 m/s to 4.5 m/s reduced the fraction of carcasses estimated to be within plots from 92% to 72% because on average, carcasses fell further from turbines with a higher cut-in speed. The TIMR treatment reduced the fraction of carcasses estimated to be within plots to at most 51%; the true fraction is unknown because our search radius was not sufficient to characterize the carcass distribution. Consequently, the true fatality rate at TIMR turbines is unknown but at least 5.0 fatalities per MW, and efficacy of the TIMR treatment is unknown, but at most 75%. This is in contrast to the 84.5% estimated by Hayes et al. [12] with the same data. Their study used different fatality estimators than we used, but the reduced curtailment efficacy reported here is due almost entirely to the addition of the area correction, confirming Huso’s [17] contention that “reduced detectability of fatalities occurring during higher wind speed conditions may bias results in favor of the curtailment treatment.” The increased variability in our fatality estimates (Table 3), particularly for the TIMR treatment, could be ameliorated by using a larger search radius, as is common in current post-construction monitoring practice (for example, see May et al. [29] and Weaver et al. [30]). The fraction of carcasses estimated to occur within control plots (92%) is in good agreement with the 89% estimated by Gruver et al. [18] that informed the study design, though the estimation methods used there were different than the methods we used here.

**Fig 2:**
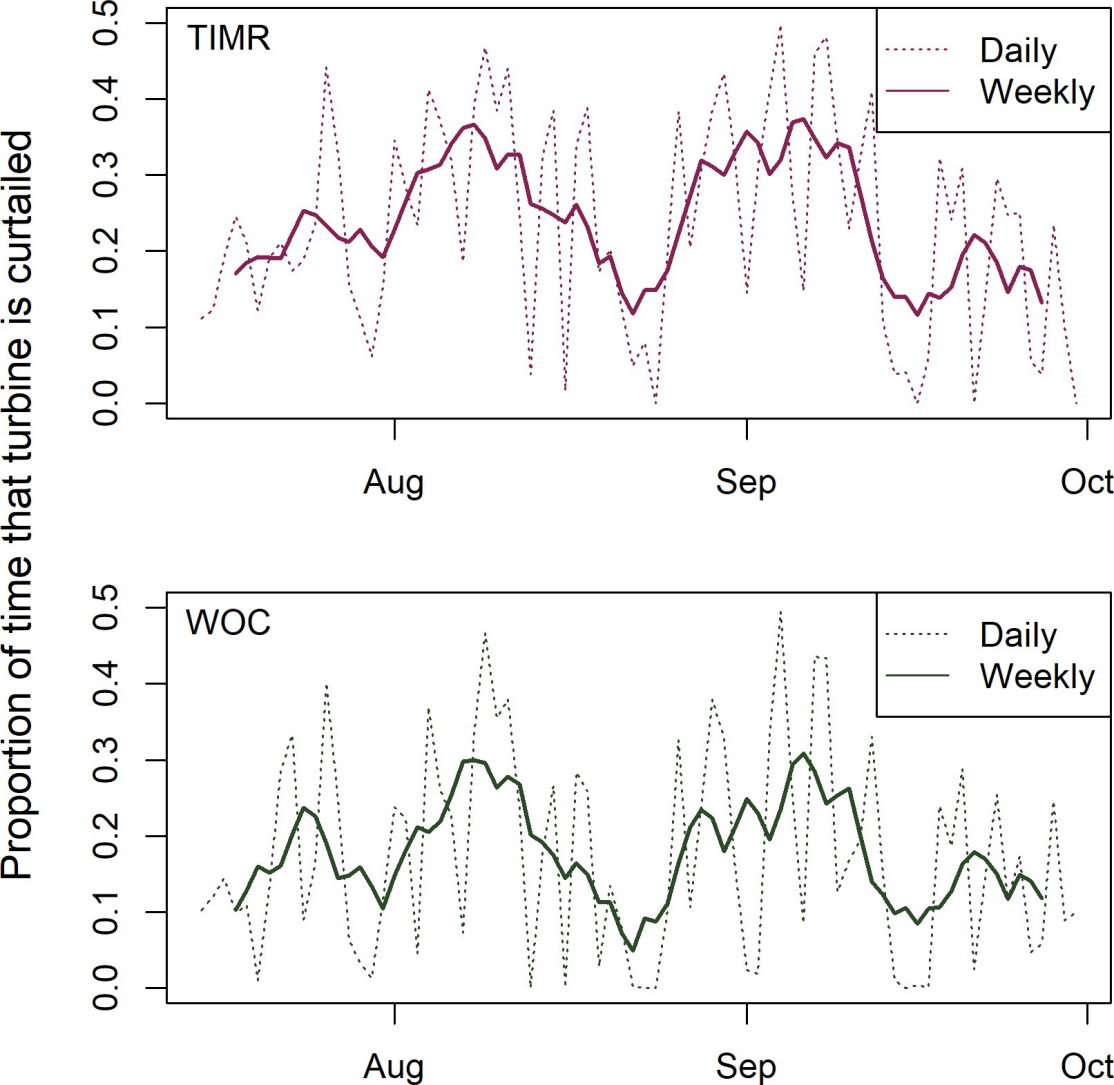
Proportion of time that study turbines were curtailed at the Blue Sky Green Field Wind Energy Facility, Fond du Lac County, Wisconsin, from July 15 − September 30, 2015. Data were collected at 10 Turbine Integrated Mortality Reduction (TIMR) system-curtailed turbines (top panel) and at 10 wind speed-only curtailed (WOC) turbines with a cut-in speed of 4.5 m/s (bottom panel). Control turbines (not shown) were not curtailed at all. Proportion of time curtailed are presented on a nightly basis and on a rolling 7-day average basis.

We did not have access to the acoustic data that informed the proprietary TIMR control system, nor would the suppliers of the TIMR control system provide information about calibration procedures and sensitivity tests (if any) conducted on the acoustic detectors. Consequently, we cannot warrant that the system will perform similarly in future deployments. We did have access to the BSGF turbine operation data, which records rotor revolutions per minute and wind speeds at each turbine in 10-minute intervals. These data show that proportion of time turbines in the TIMR treatment were curtailed was generally similar to the proportion of time turbines in the WOC treatment were curtailed, throughout the season (Fig 2). Although we cannot conclusively demonstrate that the acoustic detectors associated with the TIMR treatment did not experience drifting sensitivity—and thus a drifting treatment effect, we are confident that the TIMR system produced a treatment effect throughout the study period.

**Fig 3:**
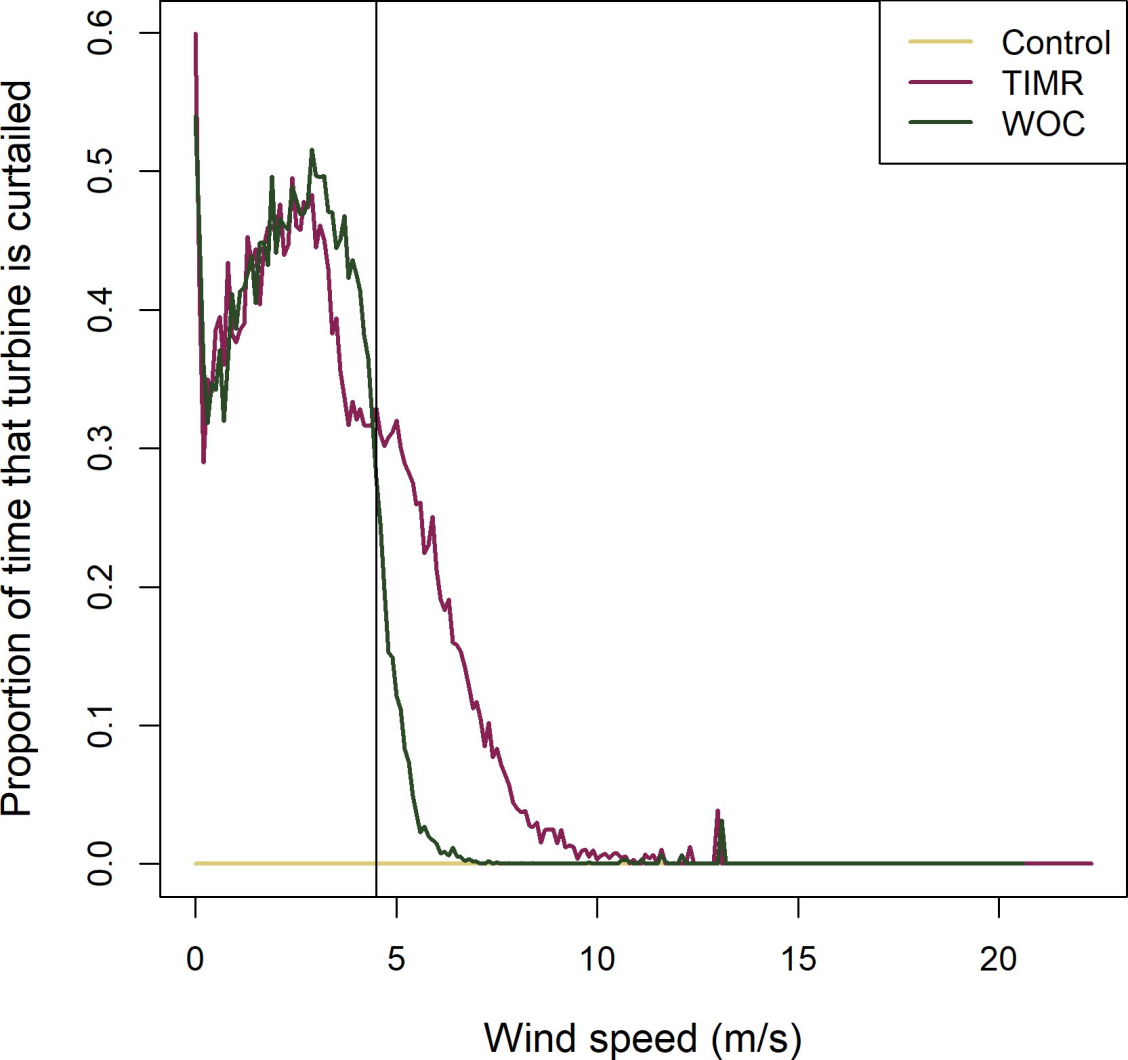
Proportion of time that study turbines were curtailed, by wind speed, at the Blue Sky Green Field Wind Energy Facility, Fond du Lac County, Wisconsin, from July 15 − September 30, 2015. Data were collected at 10 control turbines (Control), 10 wind speed-only curtailed (WOC) turbines with a cut-in speed of 4.5 m/s, and 10 Turbine Integrated Mortality Reduction (TIMR) system-curtailed turbines.

Turbines in the TIMR treatment at the BSGF were curtailed only when bat activity had been detected within the previous 30 minutes. Curtailment occurred for 39.4% of nighttime hours during the study period compared to 31.0% for the WOC turbines, but curtailment periods included wind speeds up to 8.0 m/s (Fig 3) and greater wind speeds produce more energy. Although much of the TIMR-triggered curtailment occurred when wind speeds were below 4.5 m/s, coinciding with curtailment of WOC turbines, other curtailment events occurred at wind speeds greater than the WOC threshold. The Vestas V82 turbine model used at the BSGF has a cut-in speed of 3.5 m/s and achieves peak energy production at 13.5 m/s [20], at which point energy production remains steady regardless of increasing wind speed. The turbines’ power curve increases rapidly between 3.5 and 13.5 m/s wind speeds, resulting in 280% higher production losses for the TIMR turbines even though the proportion of time turbines were curtailed was only modestly higher than for the WOC turbines.

Power and revenue losses due to TIMR-informed curtailment may be less pronounced at wind energy facilities with higher wind regimes. Average wind speed at the BSGF during the study period was 5.6–5.8 m/s. The BSGF is located in an area with low wind regimes (Wind Power Class 1–2) that is classified as “marginal for utility-scale applications” [31, 32]. Other areas of the US have Wind Power Classes of up to 7, with corresponding average wind speeds of up to 11.9 m/s [31, 32]. Facilities located in areas with higher wind regimes may have fewer TIMR-triggered curtailment events because wind speeds would be more frequently above the threshold for the TIMR system to engage.

It is unlikely that there is a single optimal wind speed threshold for TIMR; the optimum for each wind project depends on the need to reduce bat fatalities, the capacity of the facility to absorb production losses, bat activity at the site, and the wind regime at the site. Wind energy production facilities will need to weigh these factors on a site-by-site basis. For example, a typical avoidance strategy utilized in conservation planning documents for US wind energy facilities that pose a risk to federally listed bat species is to implement a seasonal WOC strategy when wind speeds of 6.9 m/s or less occur [33]. In such cases, using TIMR with an upper wind speed threshold of 6.9 m/s may provide acceptable levels of protection to bats and also allow turbines to operate when bats are absent from facility, thus reducing economic losses. Studies that compare WOC and TIMR using similar cut-in speeds will be useful to understand to what extent TIMR sacrifices efficacy to reduce production losses associated with WOC. Future studies will benefit by ensuring adequate plot size and accounting for carcass spatial distributions appropriately. Our results suggest that the TIMR system is effective at reducing bat fatalities, but additional studies will clarify the cost-benefit tradeoff associated with the TIMR system.

## Supporting information

S1 File Minimal data set to reproduce the analyses. (ZIP)

S2 File Hayes et al. 2019 publication based on a subset of our data. (PDF)
