# Supplementary material for: Efficacy and cost of acoustic-informed and wind speed-only turbine curtailment to reduce bat fatalities at a wind energy facility in Wisconsin
Source: PLoS One. 2022 Apr 8;17(4):e0266500. doi: 10.1371/journal.pone.0266500 (PMC8992975; doi:10.1371/journal.pone.0266500)
Supplement: S2 File — (PDF) [file pone.0266500.s002.pdf]

Ecological Applications, 0(0), 2019, e01881
© 2019 by the Ecological Society of America

A smart curtailment approach for reducing bat fatalities and curtailment time at wind energy facilities

Mark A. Hayes [1,8] Lauren A. Hooton,[2] Karen L. Gilland,[1] Chuck Grandgent,[1] Robin L. Smith,[1] Stephen R. Lindsay,[1] Jason D. Collins,[3] Susan M. Schumacher,[4] Paul A. Rabie,[5] Jeffrey C. Gruver,[6] and John Goodrich-Mahoney[7]

[1] *Normandeau Associates, Gainesville, Florida 32609 USA*
[2] *Trent University, Peterborough, Ontario K9J 0G2 Canada*
[3] *Normandeau Associates, Stowe, Pennsylvania 19464 USA*
[4] *We Energies, Milwaukee, Wisconsin 53203 USA*
[5] *Western Ecosystems Technology, Laramie, Wyoming 82070 USA*
[6] *Rocky Mountain Bat Conservancy, Laramie, Wyoming 82070 USA*
[7] *Electric Power Research Institute, Palo Alto, California 94304 USA*

Citation: Hayes, M. A., L. A. Hooton, K. L. Gilland, C. Grandgent, R. L. Smith, S. R. Lindsay, J. D. Collins, S. M. Schumacher, P. A. Rabie, J. C. Gruver, and J. Goodrich-Mahoney. 2019. A smart curtailment approach for reducing bat fatalities and curtailment time at wind energy facilities. Ecological Applications 00(00):e01881. 10.1002/eap.1881

Abstract. The development and expansion of wind energy is considered a key global threat to bat populations. Bat carcasses are being found underneath wind turbines across North and South America, Eurasia, Africa, and the Austro-Pacific. However, relatively little is known about the comparative impacts of techniques designed to modify turbine operations in ways that reduce bat fatalities associated with wind energy facilities. This study tests a novel approach for reducing bat fatalities and curtailment time at a wind energy facility in the United States, then compares these results to operational mitigation techniques used at other study sites in North America and Europe. The study was conducted in Wisconsin during 2015 using a new system of tools for analyzing bat activity and wind speed data to make near real-time curtailment decisions when bats are detected in the area at control turbines ($N = 10$) vs. treatment turbines ($N = 10$). The results show that this smart curtailment approach (referred to as Turbine Integrated Mortality Reduction, TIMR) significantly reduced fatality estimates for treatment turbines relative to control turbines for pooled species data, and for each of five species observed at the study site: pooled data (–84.5%); eastern red bat (*Lasiurus borealis*, –82.5%); hoary bat (*Lasiurus cinereus*, –81.4%); silver-haired bat (*Lasionycteris noctivagans*, –90.9%); big brown bat (*Eptesicus fuscus*, –74.2%); and little brown bat (*Myotis lucifugus*, –91.4%). The approach reduced power generation and estimated annual revenue at the wind energy facility by ≤ 3.2% for treatment turbines relative to control turbines, and we estimate that the approach would have reduced curtailment time by 48% relative to turbines operated under a standard curtailment rule used in North America. This approach significantly reduced fatalities associated with all species evaluated, each of which has broad distributions in North America and different ecological affinities, several of which represent species most affected by wind development in North America. While we recognize that this approach needs to be validated in other areas experiencing rapid wind energy development, we anticipate that this approach has the potential to significantly reduce bat fatalities in other ecoregions and with other bat species assemblages in North America and beyond.

Key words: Chiroptera; operational mitigation; ReBAT; smart curtailment; turbine integrated mortality reduction; wind energy development; Wisconsin.

Introduction

The broad adoption of wind-generated power into the global energy portfolio has the potential to substantially decrease carbon and greenhouse gasses emitted into the atmosphere by humans and help build environmentally sustainable economies (DeCarolis and Keith 2006, Chu and Majumdar 2012, IPCC 2014, Jacobson et al. 2015). Despite the promise of wind energy, however, some stakeholders have expressed concerns that wind energy facilities can have negative impacts on individual animals, wildlife populations, species, and ecosystems (Kuvlesky et al. 2007, Saidur et al. 2011, Sánchez-Zapata et al. 2016, Banerjee et al. 2017, Gasparatos

Manuscript received 18 May 2018; revised 20 December 2018; accepted 1 February 2019. Corresponding Editor: Ernie Valdez.
[8] E-mail: mhayes@normandeau.com

et al. 2017, Gibson et al. 2017, Zerrahn 2017). For example, some bird species have been impacted by wind energy facilities, including songbirds, raptors, waterfowl, and other species (Drewitt and Langston 2006, Lovich and Ennen 2013, Katzner et al. 2016). Bat ecologists have also expressed concerns about the impacts of wind energy development on bats (Kunz et al. 2007, Arnett et al. 2008, 2016, Cryan and Barclay 2009, Arnett and Baerwald 2013, Alverez and Lidicker 2015, Hein and Schirmacher 2016). In North America, dead bats have been observed at most operational wind energy facilities where data are available (Arnett and Baerwald 2013, Arnett et al. 2016, Zimmerling and Francis 2016), and some researchers in North America have concluded that fatalities resulting from interactions with wind turbines are potentially resulting in tens to hundreds of thousands of fatalities annually (Cryan 2011, Arnett and Baerwald 2013, Hayes 2013, Smallwood 2013, Frick et al. 2017). Furthermore, recent population dynamics modeling suggests that some species, such as North American hoary bats (*Lasiurus cinereus*; Frick et al. 2017), may be at significantly increased risk for population extinctions due to wind energy development. Ecologists in other geographic areas have also observed substantial bat fatalities associated with wind energy facilities, including in México, Central and South America (Bernard et al. 2014, Rodríguez-Durán and Feliciano-Robles 2015); Eurasia (Rydell et al. 2010, Amorim et al. 2012, Voigt et al. 2012, 2016, Lehnert et al. 2014); Africa (Doty and Martin 2013, Aronson et al. 2014); Australia, New Zealand, and the Pacific Islands (Gorresen et al. 2008, Hull and Cawthen 2013); among other locations (see Arnett et al. 2016 for a comprehensive global review). These observations imply that the impacts of wind energy facilities on bat populations are not constrained to North America and Europe, where most research has occurred, but may impact bat populations globally.

Concerns over the past decade about the impacts of wind energy development on bat populations and species have led to an improved understanding of where and when various bat species are most susceptible to negative interactions at wind energy facilities. At the local scale, analyses of bat behavior and occurrence patterns, along with radar imagery and videography of bats flying at and near wind energy facilities, has improved our understanding of how bat behavior is influenced by wind turbines and rotating turbine blades (Horn et al. 2006, Cryan 2008, Weller and Baldwin 2012, Cryan et al. 2014*a*, Jameson and Willis 2014). On regional and continental scales, use of stable isotope analyses, wildlife tracking, and various approaches to geospatial modeling have led to an improved understanding of the seasonal movement patterns and distributions of migratory species, along with information related to when and where these species might be most susceptible to negative interactions at wind energy facilities (Santos et al. 2013, Cryan et al. 2014*b*, Roscioni et al. 2014, Hayes et al. 2015, Rodhouse et al. 2015, Plyant et al. 2016, Weller et al. 2016). Additionally, observational and manipulative studies at wind energy facilities have led to an improved understanding of how these facilities can be operated in ways that reduce bat fatalities (Arnett and Baerwald 2013, Arnett et al. 2016). For example, it has been proposed that bats tend to be more active during periods associated with moderate and low wind speeds (Kerns et al. 2005), and thus may be at more risk around wind turbines when wind speeds are lower (for reviews of this literature, see Arnett and Baerwald 2013, Arnett et al. 2016).

A number of studies have demonstrated that modifying nighttime wind energy facility operation so that turbine blades are only allowed to rotate very slowly and are thus not a substantial danger to bats, or in some other way modified when the late summer and autumn migratory period coincides with lower wind speeds, can substantially reduce bat fatalities (Behr and von Helversen 2006, Baerwald et al. 2009, Arnett et al. 2011, Beucher et al. 2011, Young et al. 2011, Good et al. 2013, Martin 2015, Martin et al. 2017). These modifications in turbine operation aimed at reducing bat fatalities are generally referred to collectively as operational mitigation (USFWS 2012, Arnett and Baerwald 2013). Those approaches expected "to reduce to the smallest practicable amount or degree" are referred to as minimization (U.S. Fish and Wildlife Service 2012:61); in this paper we use the term "mitigation" ("avoiding or minimizing significant adverse impacts…" (U.S. Fish and Wildlife Service 2012:61), because it is not yet clear to us the magnitude of possible reductions in fatalities at wind energy facilities.

Operational mitigation can be viewed as part of a mitigation hierarchy (Kiesecker et al. 2009, Marques et al. 2014, Peste et al. 2015) of possible approaches to avoid, minimize, and compensate for the impacts of wind energy facilities on bat populations and species. Mitigation strategies have successfully reduced bat fatalities observed at wind energy facilities and have been incorporated into key regulatory guidance documents in North America and Europe (USFWS 2012, Rodrigues et al. 2014). For example, a common operational mitigation strategy in North America is to curtail by pitching the blades, rotating the blades out of the prevailing wind, which causes them to rotate slowly (less than one full rotation per minute), when wind speeds are below a certain threshold (e.g., 5.0 or 6.5 m/s) and bats are more likely to be present. These strategies are generally referred to as "blanket curtailment" when all turbines in a wind facility are curtailed under certain wind conditions with the intent of reducing bat fatalities (T. Allison, *personal communication*). However, mitigation strategies that require wind energy facilities to curtail wind turbines when bats are most at risk, such as at night from mid-summer through late autumn, tend to coincide with periods of high electrical demand (Gripe 2004, Jacobson et al. 2015) and can reduce the amount of energy that a

wind energy facility can produce during these periods. In an attempt to reduce bat fatalities while optimizing energy production, research has begun to focus on the development of models to predict when bats might be at most risk at a given wind energy facility, and in automated monitoring of bats at wind energy facilities (Arnett et al. 2016). For example, curtailment algorithms using correlative statistical models are being used in Europe to make predictions about the level of risk to bats at wind energy facilities under various environmental conditions, and to guide curtailment decisions (Korner-Nievergelt et al. 2013, Hanagasioglu et al. 2015, Behr et al. 2017). When compared to blanket curtailment approaches, these predictive modeling approaches have substantial promise in helping to reduce bat and other wildlife fatalities while increasing operational flexibility (e.g., allowing the turbines to continue producing energy when they might otherwise be curtailed based only on wind speed information) and energy production at wind energy facilities (Behr et al. 2017). However, application of statistical risk models across larger geographic areas (such as at the regional and continental scales) and among a variety of bat species assemblages may be much more challenging, and may require many years of data to sufficiently train models, even within a given ecoregion (Voigt et al. 2015, 2016). Other approaches to reducing bat fatalities at wind energy facilities have focused on technological advances in real-time acoustic and video monitoring of bats and birds at these facilities (Willmott et al. 2015) and using electromagnetic and ultrasonic signals and deterrents to reduce bat activity near turbines (Nicholls and Racey 2007, 2009, Szewczak and Arnett 2007, Arnett et al. 2013, Gorresen et al. 2015, Cryan et al. 2016). These technological advances also suggest substantial promise, but may not always be applicable across species assemblages in a variety of ecoregions (Arnett et al. 2016).

Despite these advances in our understanding of bat ecology and behavior, along with the development of techniques and technologies for reducing bat fatalities at wind energy facilities, some stakeholders and wind energy producers have expressed an interest in finding ways to reduce bat fatalities while increasing the time during which turbines continue operation and power production relative to standard operational mitigation approaches (AWWI 2017, Martin et al. 2017). These stakeholders have also expressed an interest in so-called "smart curtailment" approaches that use and combine information about real-time bat activity and environmental information, such as weather conditions, and which might be generalizable across large spatial areas (such as within and among ecoregions and continents) and species assemblages. Some of the goals of smart curtailment strategies are to improve power production and economic performance at wind energy facilities, while reducing impacts on bats and their populations; improve regional grid reliability (including at the fine temporal scale of seconds to minutes); contribute more to green energy sustainability goals and benchmarks through reductions in use of carbon-intensive energy sources (Jacobson et al. 2015), including during high use periods; and reduce the financial and regulatory uncertainty associated with wind energy facility development and operation (AWWI 2017).

Here, we describe a controlled experiment to test a smart curtailment approach for reducing bat fatalities at wind energy facilities and reducing curtailment time when compared to blanket curtailment techniques. This program of research and technology development has six key ongoing goals: (1) use real-time bat acoustic monitoring to provide near real-time feedback (e.g., within seconds to minutes) about bat activity to a wind energy facility's supervisory control and data acquisition (SCADA) unit, which can in turn be used to curtail turbines only when active bats are detected in the area and the risk level is considered high; (2) significantly reduce bat fatalities of all bats when compared to normally operated turbines; (3) significantly reduce migratory bat fatalities, including fatalities associated with species in the *Lasiurus* and *Lasionycteris* genera, which have been substantially impacted by wind energy development in North America (Arnett et al. 2016, Frick et al. 2017); (4) significantly reduce *Myotis* fatalities, which is the most speciose genus in the United States and Canada, and populations of some species are in decline due to bat white-nose syndrome (Frick et al. 2010); (5) reduce curtailment time relative to blanket curtailment by allowing turbines to continue operation when bats are not detected; and (6) develop a smart curtailment approach that has the potential to be generalizable and useful in a variety of geographic regions, wind speed regimes, and with different resident and migratory bat assemblages. We view this project from an adaptive management perspective (Williams 2011) and strive to recognize and articulate the uncertainties associated with this smart curtailment approach and the long-term dynamics of bat populations with continental-scale distributions, some of which also migrate on continental scales. Thus, this project is part of a collaborative, long-term research program aimed at understanding the strengths and weaknesses of various strategies for reducing bat fatalities at wind energy facilities.

Methods

Study area

This study took place at the Blue Sky Green Field Wind Energy Center, Fond Du Lac County, Wisconsin (hereafter BSGF; Fig. 1). This is an ~42 km^2 (4,200 ha) wind energy production site operated by We Energies. BSGF provides wind-generated electricity to consumers in Wisconsin and Michigan's Upper Peninsula region. Construction of the facility was initiated in 2007 and production began in 2008. BSGF consists of 88 monopole Vestas Wind Systems V82 turbines (Vestas 2005).

Each turbine is 80 m tall, with 41 m blades and a rotor-swept area of 5,281 m^2, and maximum height of 121 m (Vestas 2005). The V82 model turbines initiate power generation of 0.25 MW at a cut-in speed of 3.5 m/s (Vestas 2005); this is the wind speed at which the generator is engaged and the turbine begins providing power to the electric grid (Wagner and Mathur 2013). This model is feathered and blades rotate slowly below the cut-in speed. As wind speed increases, power generation increases to a maximum production of 1.65 MW at 13.4 m/s (Vestas 2005 this value is sometimes referred to as "nameplate rating" or "rated power"). The turbines have a cut-out wind speed of 24.1 m/s (Vestas 2005, Sutter and Schumacher 2017); this is the speed at which power generation is discontinued by rotating the blades out of the prevailing wind to prevent damage to the turbine (Wagner and Mathur 2013). Of the 88 turbines at the BSGF site, 20 were representatively allocated to control and treatment groups, with 10 turbines in each group (Fig. 1); once a turbine was selected for the treatment or control group it stayed in that group for the duration of the study. Turbines were selected for control and treatment groups using a systematic design using a random starting point and adjusted as necessary depending on landowner participation. Due to landowner constraints the turbines could not be allocated to groups in a formally random way. Treatment and control turbines were spatially paired such that pairs of control and treatment turbines were near each other and control and treatment turbines were allocated throughout the BSGF facility.

The BSGF site is situated between Lake Winnebago and Lake Michigan near the edge of the Niagara Escarpment in the Southeastern Wisconsin Savannah and Till Plain ecoregion (Omernik et al. 2000). Elevation at turbines ranges from about 240 to 335 m above sea level. The principal land use in the area is agriculture, interspersed with woodlands and wetlands. The area experiences a humid continental climate with hot summers (mean high temperature in July ~27.4°C), cold winters (mean low temperature in January –12.6°C), and about 795 mm average annual precipitation Midwestern Regional Climate Center using the Chilton, Calumet County, Wisconsin climate summary data; data *available online*).[9] BSGF has a relatively low wind speed regime when compared to some other parts of North America, which has the potential to make the site more dangerous for bats and also exposes wind developers and investors to slower returns on investment. The average annual wind speed at BSGF is 6.3 m/s, with substantial proportions of power generation occurring between 3.5 and 8.5 m/s (Sutter and Schumacher 2017). Wind speed varies by season and time of day, with average wind speeds tending to be lowest in July and August, then increased in September and October and throughout much of the rest of the year, with warmer season wind speeds tending to be higher at night (Sutter and Schumacher 2017). The BSGF facility has a capacity factor of 26.9%; this is the ratio of average annual power production compared to the peak potential production for the combined wind turbines at the facility. The BSGF capacity factor is in the lower end of capacity factors for the United States (~26–51%; Sutter and Schumacher 2017).

Seven bat species are known to occur in southeastern Wisconsin near the BSGF site (Kurta 2017): little brown bat (*Myotis lucifugus*), northern long-eared bat (*M. septentrionalis*), big brown bat (*Eptesicus fuscus*), tricolored bat (*Perimyotis subflavus*), silver-haired bat (*Lasionycteris noctivagans*), hoary bat (*Lasiurus cinereus*), and eastern red bat (*Lasiurus borealis*) (data *available online*).[10] The *Myotis*, *Eptesicus*, and *Perimyotis* species are either known to, or likely to, hibernate in areas near summer habitat and are not known to migrate long distances between summer and winter habitat (Kurta 2017). These species are likely to occur throughout the year, with activity levels lowest during the winter hibernation period and highest during the spring, summer, and autumn. The three Lasiurine species (*L. noctivagans*, *L. cinereus*, and *L. borealis*) tend to undertake seasonal migrations and are known to move long distances between summer and winter grounds (Cryan 2003, Cryan et al. 2014*b*, Weller et al. 2016). Lasiurine species occur most frequently among bat carcasses found underneath wind turbines in North America (Arnett et al. 2016). Summer and winter bat roosting habitat occurs in various areas in southern Wisconsin, and a hibernation site for cave-dwelling bats, such as *Myotis* species, is located within 25 km of the wind energy facility (e.g., Neda Mine).

Acoustic and wind data collection and analysis

Acoustic detectors for recording and transmitting bat acoustic data (ReBAT) and a Turbine Integrated Mortality Reduction (TIMR) systems (Normandeau Associates, Gainesville, Florida, USA) were used to detect, record, transmit, and analyze bat sound information. ReBAT systems consist of hardware and software designed for detecting, filtering, recording, and transmitting full spectrum bat acoustic data in remote conditions, and can be mounted on the nacelles of wind turbines. Four ReBAT systems were mounted on turbine nacelles. One ReBAT system was mounted on a treatment turbine, one ReBAT system was mounted on each of two control turbines, and one ReBAT system was mounted on a turbine that was not included in study (Fig. 1). The four ReBAT systems were deployed so that they sampled the airspace above and below each ReBAT system at turbines in the northern, northwestern, southwestern, and southeastern parts of the wind energy facility; these are the directions from which bats would be expected to be flying given the prevailing wind regime in the area. Each

[9] https://mrcc.illinois.edu/mw_climate/climateSummaries/climSumm.jsp

[10] https://github.com/mark-a-hayes/TIMR-species-data

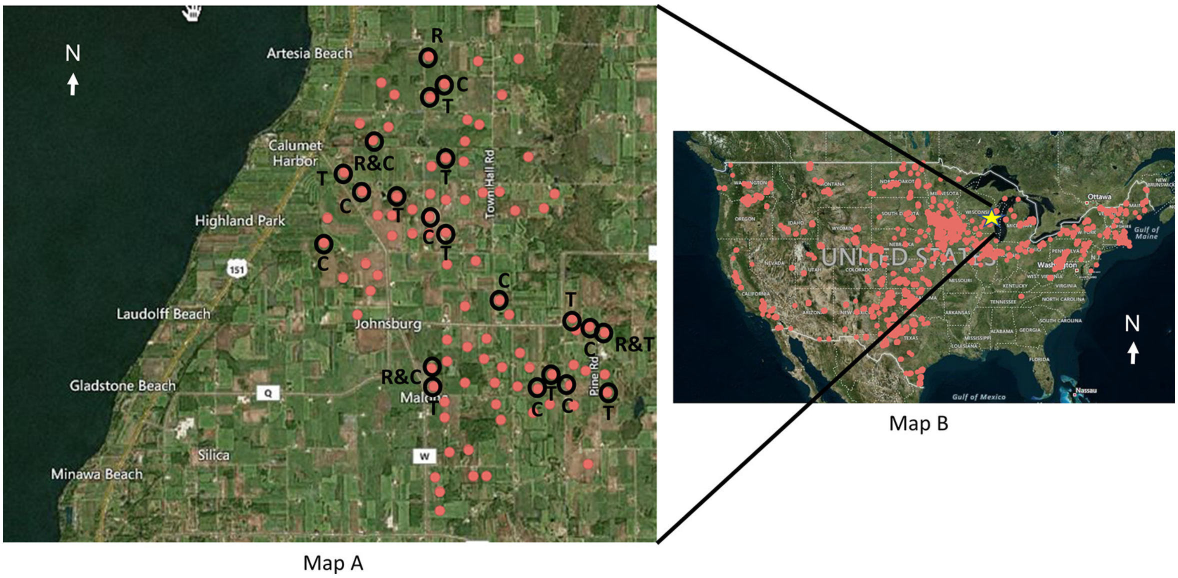

Fig. 1. The Blue Sky Green Field study site, Fond Du Lac County, Wisconsin, USA. Map A shows the locations of individual wind turbines (red dots), including turbines used in the study (circled), including control turbines (C; N = 10), treatment turbines (T; N = 10), and turbines with ReBAT acoustic systems mounted on them (R; N = 4). Map B shows the study location (yellow star) in the context of other wind energy facilities in the United States. These maps were created using the USGS Wind Farm interactive site (https://eerscmap.usgs.gov/windfarm/).

ReBAT system consisted of two acoustic detectors, one of which sampled the airspace below the nacelle and the other was fitted with a reflector plate to sample the airspace above the nacelle level. There was no obvious qualitative difference in quality of sound files recorded at detectors with and without reflector plates, or while turbines were operating vs. during curtailment. Each detector contained a directional ultrasonic sensor with a frequency range of 1–125 KHz capable of continuously sensing sounds from 0 to 90 dB intensity (SensComp 2013; Binary Acoustics Technology, Tucson, Arizona, USA). Each ReBAT system detected and fed continuous sound information to a digitizer and then a filter, which selected sound information for recording using a standardized filter setting (frequency range: 15–80 KHz), and recorded sound information in 1.7 s WAV files, along with associated metadata, including a time stamp, the sensor identification, and the ReBAT system that recorded the file. Once WAV files were created and stored in ReBAT storage, they were subjected to an algorithm designed to identify probable bat call information within sound files (SCAN'R Snapshot Characterization and Analysis software for bat call analysis; Binary Acoustics Technology, Tucson, Arizona, USA). Each ReBAT system was affixed to the side of the turbine nacelle's cooling radiator, which on this model of turbine extends across the top of the nacelle. The ReBAT systems sent all recorded sound files identified as containing probable bat call information in real-time via modems and the cellular network to the TIMR server located at the BSGF operations center. Acoustic data from the four ReBAT systems were continuously consolidated, processed, and analyzed in near real-time using automated scripts, and a network, server, and database system maintained at the BSGF operations center (see Sutter and Schumacher 2017 for full details). Subsequently, the acoustic data were retrieved daily by the Normandeau Operations Center in Gainesville, Florida, for analysis and long-term storage. The wind speed data (m/s) used was collected in 10-min increments and was provided by the BSGF SCADA to Normandeau's TIMR system. The wind speed data (m/s) for risk analysis were derived from an anemometer mounted on the nacelle of 1 turbine (B16); the central location of this turbine in the wind facility and past wind speed data suggested that this turbine represented typical wind conditions experienced at the BSGF site. The stakeholder group considered consolidating wind speed information from more than one turbine but concluded that using this turbine would be suitable for the TIMR risk model. The average wind speed for each 10-min period was used as the wind-speed measurement for the period. Wind speed data were measured and collected concurrently to acoustic data collection, and wind speed data for each turbine in the study was also collected. The optimal number and placement of ReBAT systems for a given wind energy facility and turbine model is an area of continuing research.

The smart curtailment model

A risk model was used to make continuous near real-time binary risk assessments and provide feedback to

the BSGF control facility through a SCADA interface. The risk model was applied during the mid-summer through autumn period (15 July–31 October), and daily from 18:00 to 06:00 the following morning during July and August and 18:00 to 07:00 the following morning during September and October. At all other times the risk model was not used and wind turbines operated normally. The binary risk model combined current wind speed conditions with current consolidated acoustic bat activity data using the ReBAT/TIMR system, as follows: if the measured wind speed was <8.0 m/s, and ≥1 bat call sequence was identified by the ReBAT systems in the previous 10 min, then the risk was considered high and all treatment turbines were curtailed by pitching the blades out of the wind and allowing the blades to rotate at ≤1 rpm, while control turbines were allowed to operate normally; alternatively, if the measured wind speed was ≥8.0 m/s, all turbines were allowed to operate normally without curtailment regardless of bat activity. For the purposes of this study a bat call sequence was considered a recorded WAV file with one or more sounds that passed the filter settings and SCAN'R algorithm, and thus was considered to represent probable bat sound information. Each 10-min period was considered discretely, not on a rolling time basis. The decision to use 7.9 m/s as the upper threshold for curtailment, as opposed to 5.0, 6.5, or 6.9 m/s (USFWS 2016), for example, was made after consultation with the key stakeholders. Given that, during the prior year, bat sounds were sometimes recorded at wind speeds >6.9 m/s (Sutter and Schumacher 2017), the stakeholder group decided to set the curtailment threshold at 7.9 m/s. Correlative statistical risk models were considered for use in this study (e.g., Poisson, zero-inflated, and generalized linear mixed models; see Sutter and Schumacher 2017 for full details). But because a statistical model that seemed likely to reliably predict fatality risk at this site was not immediately evident given earlier data and analysis, a statistical risk model was not used during this study. There was no difference between control and treatment operation when wind speeds were ≥8.0 m/s. The risk prediction derived from the curtailment rules was communicated every 10 min as a binary risk estimate (low or high) to the SCADA unit of the BSGF facility. Smaller and larger curtailment intervals were considered (e.g., 5 and 30 min), but the 10-min interval was considered a reasonable timeframe with which to make curtailment decisions. When the risk communicated to the SCADA unit was high, the SCADA would undertake the appropriate action for each of the treatment turbines, and would take no action for the control turbines. For the first period considered to be of high risk, the treatment turbines were curtailed for 30 min. Thereafter, if the risk was still high, the risk model would consider returning to normal operation every 10 min and would do so once a low-risk prediction was received. All time periods were considered discretely, not on a rolling time basis.

Carcass searches and fatality analysis

Carcass searches.—The 20 turbines in the study were searched once daily for bat and bird carcasses by trained technicians between 1 June and 31 October 2015 (Gruver et al. 2016). The search plots were square (80 by 80 m) and were established such that the turbine tower was centered on the plot. During prior carcass monitoring at this wind energy facility (Gruver et al. 2016) ~90% of carcasses fell within 40 m of turbine towers. A carcass "fall distribution" study was not conducted during this study, so the distribution of control and treatment carcasses is not fully understood. We recommend inclusion of a fall distribution study in future studies. Transects spaced 5.0 m apart were established in each plot (80 m transect, 5 m between transects, 16 transects per plot). Technicians walked transects at ~45–60 m/min while searching the ground for bat and bird carcasses. While walking transects technicians strived to search 3 m on either side of the transect line so that the search area for each new transect overlapped with the previous transect. If a transect was along the edge of a plot, the searcher searched in the same manner as for interior transects, but if carcasses were found outside a plot they were not included as carcasses discovered within the plot. To maintain relatively consistent and easily searchable ground cover within and among plots, vegetation was mowed regularly and herbicide applications were applied as needed during the course of the study. Fine-scale data related to vegetation cover and height on search plots was not collected; we encourage collection and use of such data in future studies. It was assumed that the ground cover on all plots was approximately equivalent. Carcass searches were initiated in the morning with the first plot search occurring as soon as practical after sunrise. Technicians typically required <30 min of search time per plot (16 transects × 80 m/transect = 1,280 m per plot; thus, 1,280 m/52.5 m/min = 24 min/plot); the time a technician spent on a plot varied depending on the number of carcasses found and the time necessary to record data and photograph carcasses. Plots were searched daily during the study period. The plots were allocated to a rotating search schedule so that plots were searched by different searchers at different times of day throughout the study period. Technicians logged search information on standardized forms, including date and time of carcass discovery; the species identity of each carcass; the age and sex, if known; the geographic coordinates where the carcass was found; the distance and bearing from the turbine tower; the condition of the carcass (intact, scavenged, etc.); the approximate age in days of the carcass (fresh carcasses were identifiable from the physical characteristics of the carcass, such as the shape and opacity of the eyes, etc.); and other comments as necessary. Carcasses were photographed where they were found. Safety procedures related to adverse weather and lightning were followed during carcass searches. All carcasses were identified using a unique

code, removed from the plot, and stored in a freezer on the BSGF study site. For the purposes of this analysis we assumed that most carcasses would land within the search plots, and the number of carcasses discovered in control and treatment plots was compared. We did not attempt to develop fatality estimates for the entire wind energy facility, but rather focused on estimated fatalities that were available for detection in control vs. treatment plots. Likewise, searcher and turbine data that may be useful in modeling bat fatality at these wind turbines was not used in this analysis (such as searcher identify, search time, weather conditions, and influence of missed searches and turbine maintenance); we recommend collection and consideration of data associated with these and similar covariates in future studies.

Because turbines were allocated to control and treatment groups in a way that was spatially balanced and included a random starting location, we assumed that local environmental factors and land-use patterns with the potential to influence bat activity around wind turbines, and thus fatality rates at individual turbines, would tend to be absorbed across the control and treatment groups and were not likely to bias one group against the other. Examples of these possible affects are proximity to the perimeter of the wind energy facility; proximity to preferred commuting corridors, foraging areas, and roosting resources; local insect diversity and density; variability in wind regime and environmental conditions across the wind energy facility; proximity to roads and highways; proximity to water resources; maintenance issues related to turbines that resulted in one or more turbine being inactive for a short period of time; land-use histories and patterns associated with a given property, including the crop grown, land-use, geological substrate, and/or soil type; historical applications of herbicides and pesticides on crops and vegetation; proximity to small communities, towns, and natural areas; variation in elevation across the study area; and/or temporal variability in local environmental factors, growth of vegetation, biomass, insect density and diversity, and other factors that changed on local scales over the course of the study. Although these variables were not considered during this study, we recommend consideration and possible inclusion of these and similar variables in future research.

Fatality analysis.—The daily carcass search results were compiled into a dataset for analysis. This included the species identity of each carcass found at turbines each day from 15 July to 31 October 2015 (109 d). Prior to final analysis, and because of very low bat activity levels and low carcass counts in October, the final data set was truncated to include the period from 15 July to 30 September 2015 (78 d; Sutter and Schumacher 2017). The total number of carcasses for each species found each day at the 10 control and 10 treatment turbines was also compiled, and this data set represented the data frame on which analysis was carried out. This data frame consisted of a vector for control and treatment turbines for pooled data and for each of six species for which carcasses were found during the study (*Lasiurus borealis*, *L. cinereus*, *Lasionycteris noctivagans*, *Myotis lucifugus*, *Eptesicus fuscus*, and *Perimyotis subflavus*).

Three fatality estimators were used to estimate the total number of carcasses that were available for detection in the search plots associated with control and treatment turbines during the study: the Erickson et al. (2004) estimator, the Huso (2011) estimator, and the Korner-Nievergelt et al. (2011) estimator. These fatality estimators derive estimates of the number of carcasses available for detection based on data from carcass searches and estimates of searcher efficiency and carcass persistence rates. Initially, just the Huso estimator was considered for final fatality estimation, which has been a common practice in the United States. However, some fatality estimators are based on different underlying conceptual models and treat new and old carcasses differently (Huso [2011] compared to Korner-Nievergelt et al. [2011]). Simulation results (Korner-Nievergelt et al. 2011; Figs. 1 and 2) suggested to these researchers that some estimators, including the Huso estimator, can be biased under certain conditions, such as with short search intervals and relatively high carcass persistence rates, as experienced at BSGF. Thus, given that it was unclear to some members of our group which fatality estimator would result in unbiased and the most precise fatality estimates, the three fatality estimators were used in this study, each viewed as representing an empirically derived estimate of the carcasses available for detection inside search plots in the study. The fatality estimates for control and treatment turbines derived from these estimators were also consolidated into ensemble fatality estimates for the pooled data and for each species by taking the unweighted mean of the three fatality estimators; ensemble estimates are often considered when there is uncertainty about which model from a set of possible models results in non-biased and precise estimates under the conditions of the study (Clarke et al. 2009). Searcher efficiency and carcass persistence rates for this study were derived from trials conducted at the BSGF site during 2015 following standard protocols (see Gruver et al. 2016; USGS Fatality Estimator software, Data Series 729; *available online*).[11] The carcass persistence rates were estimated by fitting four distributions (exponential, log-logistic, lognormal, and Weibull) to the data and choosing the model associated with the lowest Akaike's information criterion corrected for small sample sizes (AIC_c) following the methods of Huso (2011); the model with the lowest AIC_c value was considered the best model given the data and models considered, and the daily carcass persistence estimate derived using this model was considered the best estimate, given the data available and the model set considered (Burnham and Anderson 2002). Searcher efficiency rates were estimated

[11] https://pubs.usgs.gov/ds/729/

using logistic regression (Hosmer et al. 2013) following the approach of Huso (2011). Carcass persistence and searcher efficiency rates were estimated using the Fatality Estimator software (USGS Fatality Estimator, Data Series 729; see footnote 11). Mean estimated carcass persistence and searcher efficiency rates, and 95% confidence intervals were used in fatality estimation simulations (see below).

The estimateN fatality simulation function in the R package carcass (Korner-Nievergelt et al. 2016) was then used to complete Monte Carlo fatality estimation simulations in the R statistical software environment (R Core Team 2018; version 3.5.0). For these simulations, the following parameters were used: count, the number of carcasses found over the course of the study in control or treatment plots; *f*, estimate of mean searcher efficiency (0.60); f.lower, estimate of the lower 95% confidence interval for *f* (0.421); f.upper, estimate of the upper 95% confidence interval for *f* (0.779); *s*, estimate of mean daily carcass persistence probability (0.92); s.lower, estimate of the lower 95% confidence interval for *s* (0.89); s.upper, estimate of the upper 95% confidence interval for *s* (0.95); *d*, number of days between searches (1); *n*, number of days in the study (78); arrival, discrete (meaning that carcasses are assumed to arrive simultaneously each day, not continuously over a 24-h period (as with some models in mathematical biology that use discrete assumptions, the discrete assumption used here is a recognized simplification of natural processes, and we concluded that without mathematical processes yet available to us that more closely simulated natural process, the discrete assumption was acceptable for our purposes; we also concluded that the discrete assumption was likely superior to the continuous assumption); n_{max}, maximum number of possible fatalities in control or treatment plots (600 for pooled data and 150 for individual species data); n_{sim}, number of Monte Carlo simulations (10,000). Given that we were interested in estimating the number of carcasses available for detection within search plots, we used $a = 1$ (default), which is the proportion of animals killed that fall into the searched area. Thus we constrained these simulations to considering only carcasses available for detection within search plots. Monte Carlo simulations were conducted for each of the three fatality estimators for each of the following species groups: all control or treatment carcasses pooled in one group (pooled), eastern red bats, hoary bats, silver-haired bats, big brown bats, and little brown bats. Ensemble estimates for each group were also calculated. When using the Huso estimator only fresh carcasses that were determined to have died during the prior 24 h were used; when using the Erickson et al. and Korner-Nievergelt et al. estimators all carcasses found during carcass searches were used. In total, 36 Monte Carlo simulations were performed each using 10,000 runs. Prior to proceeding with analysis, we looked for differences in the patterns of carcass distance from turbines among control and treatment turbines. We evaluated histograms of distances between these groups, and compared the proportion of carcasses in each group occurring at 5-m increments from turbines using the Wilcoxon signed-rank test (see Samuels et al. 2012 for a discussion of this test), which is a nonparametric test used when normality cannot be assumed and when the data are composed of paired vectors (control vs. treatment turbines); we assumed that search effort was equivalent

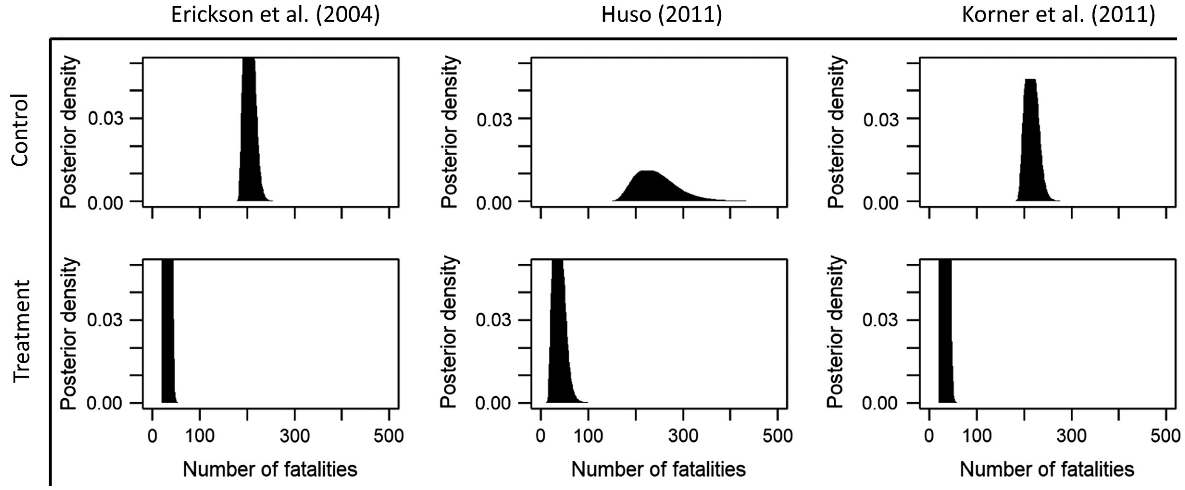

FIG. 2. Fatality estimates for pooled fatalities for all species using each of three fatality estimators (Erickson et al. 2004, Huso 2011, and Korner-Nievergelt et al. 2011) at control and treatment turbines at the Blue Sky Green Field study site, Wisconsin, USA. These are the results of 10,000 Monte Carlo simulations for each estimator and all bat species considered as a pooled group and includes eastern red bat (*Lasiurus borealis*), hoary bat (*Lasiurus cinereus*), silver-haired bat (*Lasionycteris noctivagans*), big brown bat (*Eptesicus fuscus*), and little brown bat (*Myotis lucifugus*). The results for individual species are shown in the supplementary materials. Data were collected at 10 control and 10 treatment turbines at the Blue Sky Green Field Wind Energy Center, Fond Du Lac County, Wisconsin, USA, 15 July–31 October 2015.

across distances from the turbine tower. TIMR applied the treatment when bats were detected and mean wind speed was <8.0 m/s, so the TIMR treatment could be applied at wind speeds throughout this range. Thus we did not expect the treatment to positively and significantly bias distance from the turbine towers when comparing carcasses found at treatment vs. control turbines.

As a final step, the daily differences between the number of carcasses found at control and treatment turbines were compared again using the Wilcoxon signed-rank test (Samuels et al. 2012). Normality assumptions in the control, treatment, and difference vectors for each group were checked using quantile-quantile plots (Q–Q plots) and the Shapiro-Wilk Normality Test (Crawley 2013), and each paired set of vectors failed these normality tests, as did the difference vector. We used the total number of carcasses found each day during the study at control and treatment turbines for pooled data and for individual species data. These data consisted of paired vectors comparing the daily carcasses found at control and treatment turbines for each of the 78 d. The null hypothesis of this test was that there was no difference between control and treatment populations, which can be stated as H_0: $\mu_D = 0$. For this test we used $\alpha = 0.05$. As described above for fatality estimation, we assumed in this test that random effects that were not accounted for in this study tended to be distributed among the control and treatment groups so that these effects were not likely to substantially bias the results or influence their interpretation. Our intent in using this test was to evaluate whether there was evidence for a daily difference in carcasses found at control and treatment plots.

Economic analysis

The following economic assumptions were used to estimate the revenue difference of control turbines relative to treatment turbines, which were valid during the timeframe of the 2015 study: an overnight localized margin price (LMP, in US$) of $15/MWh; a production tax credit (PTC) of $20/MWh; and a renewable energy credit (REC) of $5/MWh. This results in a gross potential market revenue of $15/MWh + $20/MWh + $5/MWh = $40/MWh (Sutter and Schumacher 2017). This gross revenue does not include potential operational changes due to unanticipated maintenance, grid restrictions, or agreements with local municipalities to reduce sound and/or shadows produced by turbine operations. During the course of the study, each turbine was monitored for average wind speed (m/s), power generation (MW), total hours of power generation (h), percent of available time that the turbine was generating power (%), hours of curtailment (h), percentage of the time the turbine was curtailed, estimated loss in generation due to curtailment (MW), and percent of the available time that the turbine was available for power generation (%). This information was used to estimate power generation and revenue for all control and treatment turbines, and the difference between power generation and revenue for these turbines when combined into control and treatment groups (Sutter and Schumacher 2017). As a final step, we estimated the percent change in curtailment time using the BSGF TIMR model compared to operating the same turbines under the U.S. Fish and Wildlife Service's technical assistance standard of blanket 6.9 m/s curtailment (U.S. Fish and Wildlife Service 2016). This economic analysis is part of ongoing research aimed at better understanding various operational mitigation and smart curtailment options and their potential economic influences on wind energy facilities under varying environmental and economic conditions; thus the economic analysis here is viewed as a foundation for ongoing comparisons to other operational mitigation strategies.

The data and code package used for the carcass and fatality analysis is available in a Zenodo repository (see *Data Availability*). This repository includes a spreadsheet summarizing fatality data, along with code in the R language used for conducting the carcass and fatality analysis. Full calculations for the economic analysis are shown in Sutter and Schumacher (2017).

Results

Between 15 July and 30 September 2015, 216 bat carcasses of six species were found inside search plots at the 20 turbines included in this study (Table 1): eastern red bats (*Lasiurus borealis*; $n = 43$), hoary bat (*Lasiurus cinereus*; $n = 57$), silver-haired bat (*Lasionycteris noctivagans*; $n = 49$), big brown bat (*Eptesicus fuscus*; $n = 34$), little brown bats (*Myotis lucifugus*; $n = 32$), and tri-colored bat (*Perimyotis subflavus*; $n = 1$; due to the low level of carcasses found fatality estimation analysis was not performed on this species). For all species the number of carcasses found at control turbines (fully operational; n = 27–48 carcasses per species) was greater than the number found at treatment turbines (smart curtailment using TIMR; n = 3–9 carcasses per species). Carcasses were found at 19 of 20 turbines, and there was no evidence that a substantially larger or smaller number of bats was found at any turbine within the control and treatment groups. Searcher efficiency was estimated as 0.600 (95% CI: 0.421–0.779), and carcass persistence was estimated as 0.92 (95% CI: 0.89–0.95; see Gruver et al. 2016 for full detail including model selection results). Fatality estimates for the number of bats available for detection within search plots tended to be similar using the Erickson et al., Huso, and Korner-Nievergelt et al. estimators (Table 1, Fig 2). In all cases the number of estimated fatalities at control plots was significantly higher vs. treatment plots and the distribution of results from Monte Carlo simulations did not overlap (Fig. 2). The unweighted ensemble mean of fatality estimates for control vs. treatment plots was significantly higher in all five species, with the change in estimated fatalities using the treatment ranging from –74.2% (big brown bats) to −91.4% (little brown bats) when compared to control

turbines (Table 1). While the Monte Carlo simulations using the three fatality estimators resulted in non-overlapping distributions of fatality estimates for the pooled data (Fig. 2), the Huso estimator tended to result in distributions with more variance (Fig. 2). The results from the Wilcoxon signed-rank tests suggest that the number of carcasses found daily at control plots tended to be significantly higher than at treatment plots in pooled data and in individual species data (Table 2).

The average wind speed measured at all turbines during the study period was 5.7 m/s. The average wind speed at control turbines (5.6 m/s) was slightly lower than for treatment turbines (5.8 m/s). All turbines in the study were available for power generation >90% of the time, with all but one turbine available >96.5% of the time (one treatment turbine was available 90.7% of the time). This reduction in availability of this turbine would tend to reduce the energy production for the treatment group while potentially reducing the number of fatalities associated with this turbine during this period of operation. We estimate that this effect could reduce energy production for treatment turbines by a maximum of 0.58% (96.5%–90.7%/10 turbines = 5.8/10 = 0.58%) and decrease fatalities by an approximately corresponding amount. In this study we were not able to identify when an individual turbine was unavailable for energy production and incorporate this information into fatality estimates. We recommend consideration of these details in future studies, along with attempts to incorporate this information into fatality estimates. Treatment operated turbines produced ~85.2% of the power and revenue that the control turbines did during the period of the study (Table 3). The 78 d during which the study took place (15 July–30 September) represented a period during which the lowest average monthly wind speeds are experienced at the BSGF site (Sutter and Schumacher 2017). Thus, given that the treatment operated turbines generated 85.2% of the power and revenue when compared to the control turbines, the TIMR smart curtailment approach used at BSGF is expected to reduce annual power generation and revenue at BSGF by (78 d/365 d/yr) × 14.8% ≤ 3.2%/yr for the year of this study. Treatment turbines were curtailed for on average 176 h between the cut-in speed of 3.5 m/s and the upper curtailment threshold of 7.9 m/s, and we estimate that turbines would have been curtailed for an average of 363 h under 6.9 m/s blanket curtailment rules (U.S. Fish and Wildlife Service 2016). Thus, curtailment time for treatment turbines was ~48.5% ((176/363) × 100) less than would have been expected under this blanket curtailment rule.

Discussion

This was the first controlled experiment to test the use of real-time bat acoustic data in combination with environmental data to guide near real-time curtailment at a wind energy facility in North America. These results

Table 1. Number of carcasses found at, and fatality estimates for, turbines used in this study.

Species group	Control turbines					Treatment turbines					
	Count	Erickson	Huso	Korner	Ensemble	Count	Erickson	Huso	Korner	Ensemble	% Δ
Pooled	187/138	205 (194, 224)	239 (184, 350)	215 (201, 239)	220 (193, 271)	29/22	32 (29, 37)	38 (27, 61)	33 (30, 40)	34 (28, 46)	−84.5
LABO	37/21	40 (37, 46)	37 (26, 59)	43 (38, 50)	40 (33, 52)	6/5	6 (6, 9)	9 (5, 18)	7 (6, 10)	7 (5, 12)	−82.5
LACI	48/40	53 (49, 59)	70 (51, 107)	55 (50, 64)	59 (50, 77)	9/7	10 (9, 13)	12 (8, 23)	10 (9, 14)	11 (8, 17)	−81.4
LANO	45/36	49 (46, 56)	63 (46, 97)	52 (47, 60)	55 (46, 71)	4/3	4 (4, 6)	6 (3, 13)	5 (4, 7)	5 (3, 9)	−90.9
EPFU	27/18	30 (27, 34)	31 (22, 51)	31 (28, 37)	31 (25, 41)	7/5	8 (7, 10)	9 (5, 18)	8 (7, 11)	8 (6, 13)	−74.2
MYLU	29/23	32 (29, 37)	40 (29, 64)	33 (30, 40)	35 (29, 47)	3/2	3 (3, 5)	4 (2, 10)	3 (3, 6)	3 (2, 7)	−91.4

Notes: Pooled, all bat species considered as a pooled group; LABO, eastern red bat (*Lasiurus borealis*); LACI, hoary bat (*Lasiurus cinereus*); LANO, silver-haired bat (*Lasionycteris noctivagans*); EPFU, big brown bat (*Eptesicus fuscus*); MYLU, little brown bat (*Myotis lucifugus*); count: first number is total number of carcasses found during the study and second number is the total number of fresh carcasses (≤24 h old); Erickson, the Erickson et al. (2004) fatality estimator; Huso, the Huso (2011) fatality estimator; Korner, the Korner-Nievergelt et al. (2011) fatality estimator; Ensemble, the unweighted average of the mean of each the three fatality estimators; % Δ, average percent change in fatality estimates when comparing treatment to control turbines using the ensemble average. Numbers in each fatality estimate cell are the mean and minimum and maximum (in parentheses) using 10,000 Monte Carlo simulations. Data were collected at 10 control and 10 treatment turbines at the Blue Sky Green Field Wind Energy Center, Fond Du Lac County, Wisconsin, USA, 15 July–31 October 2015.

TABLE 2. Results from Wilcoxon signed-rank tests for comparing carcasses found daily in this study.

Species group	*V*	*P*
Pooled	1,683	<0.0001
LABO	322	0.0001
LACI	430	<0.0001
LANO	432	<0.0001
EPFU	307	0.0025
MYLU	197	0.0004

Notes: V, critical value for the test; Pooled, all bat species considered as a pooled group; LABO, eastern red bat (*Lasiurus borealis*); LACI, hoary bat (*Lasiurus cinereus*); LANO, silver-haired bat (*Lasionycteris noctivagans*); EPFU, big brown bat (*Eptesicus fuscus*); MYLU, little brown bat (*Myotis lucifugus*). Data were collected at 10 control and 10 treatment turbines at the Blue Sky Green Field Wind Energy Center, Fond Du Lac County, Wisconsin, USA, 15 July–31 October 2015.

demonstrate how smart curtailment, combining acoustic and environmental information in near real time, can be a useful tool for reducing the impacts of wind energy facilities on individual bats and bat populations, while increasing the wind energy facility's ability to extract energy from the wind. Although collecting and analyzing acoustic data in near real-time and then communicating a risk prediction to a wind energy facility's SCADA unit requires some technological sophistication and may require associated facility-specific adaptation, once equipment and infrastructure are in place, along with a risk model to guide curtailment decisions, the approach is relatively straightforward to implement. This approach did not require prior data to train models and is not based on statistical assumptions about model structure that may or may not be reasonable given the data, ecoregion and species assemblage (Voigt et al. 2015).

This study estimates that the TIMR treatment decreased fatalities available for detection inside plots by 84.5% on average for pooled species and by 74–91% for individual species when compared to control turbines, while resulting in an annual reduction in power generation and revenue of ≤3.2%. In this study, carcasses of three migratory tree bat species (*L. borealis, L. cinereus,* and *L. noctivagans*), and three species presumed to be year-round residents of the area (*E. fuscus, M. lucifugus,* and *P. subflavus*) were found in search plots. There tended to be larger numbers of migratory tree bat species found in search plots, and slightly fewer of the resident species found (Table 1). Strikingly higher proportions of migratory tree bats, compared to *Myotis* and *Eptesicus*, have been observed at study sites in Alberta, Canada (Brown and Hamilton 2006, Baerwald et al. 2009), West Virginia (Young et al. 2011), Indiana (Good et al. 2013), and Vermont (Martin et al. 2017). The individual migratory tree bat species have different seasonal migration patterns and life histories (Cryan 2003), and the resident species in our study area are likely to move among warm and cold season roosting and foraging resources in different ways and at different times (Kurta 2017), potentially influencing susceptibility to interactions with wind turbines. It is not clear why the BSGF site would result in relatively higher fatalities for *Eptesicus* and *Myotis* species compared to some other North American sites, but this may have to do with the combined effects of population densities and natural history characteristics of these species in southern Wisconsin, potentially in combination with the relatively lower wind speed regime at the BSGF site. For example, the BSGF site is north of an abandoned mine (Neda Mine) that is known to be used as a hibernation site by large congregations of little brown bats (*Myotis lucifugus*), as well as by big brown bats (*Eptesicus fuscus*), among other species (Rupprecht 1980). It is possible that these species might be affected by nearby wind energy facilities as they transition during the autumn to this and similar hibernation sites. Some sites in Ontario, Canada have also experienced higher *Myotis* and *Eptesicus* fatalities (Zimmerling and Francis 2016), and it is possible that this part of the North American continent is an area of higher risk to these bats.

The TIMR smart curtailment approach resulted in significant reductions in fatalities across these five species, some of which almost certainly use echolocation in different ways when flying at heights that would make them susceptible to wind turbines. In terms of fatality reduction, these results compare favorably to previous research conducted at wind energy facilities in North America. Studies of operational mitigation in North America available in the literature have concentrated in Alberta, Canada, and in the mid-western and eastern

TABLE 3. Comparison of power generation and estimated revenue for the turbines used in this study.

	Power Generation (MW)			Revenue (US$)		
Operation group	Total	Per turbine	Theoretical	Total	Per turbine	Theoretical
Control	6,063	606	53,358	242,538	24,254	2,134,334
Treatment	5,165	516	45,456	206,620	20,662	1,818,252
Difference	898	90	7,902	35,918	3,592	316,082
% Control	85.2	85.2	85.2	85.2	85.2	85.2

Notes: MW, megawatt; Total, total for the 10 turbines in a group (control or treatment); Per turbine, average per turbine in a group; Theoretical, estimated value for all 88 turbines at the BSGF site during the study; Difference, absolute value of Control − Treatment values; % Control = (Treatment/Control) × 100. All values are rounded to the nearest MW or dollar. Data were collected at 10 control and 10 treatment turbines at the Blue Sky Green Field Wind Energy Center, Fond Du Lac County, Wisconsin, USA, 15 July–31 October 2015.

United States. Foundational research occurred in southeastern Alberta (Brown and Hamilton 2006, Baerwald et al. 2009). These studies evaluated the influences of braking and locking turbine blades at low wind speeds (Brown and Hamilton 2006), in altering cut-in speeds, changing the pitch angle of the blades, and feathering turbine blades (rotating the turbine blades to 90° and rotating so they are parallel with the prevailing wind direction; Baerwald et al. 2009, Arnett et al. 2016), which resulted in fatality reductions of 50–60% at treatment turbines. Arnett et al. (2011) conducted extensive analysis of bat fatalities at a wind energy facility in Pennsylvania and showed that increasing cut-in speeds from 3.5 to 5.0 and 6.5 m/s reduced bat fatalities by 44–93%. Young et al. (2011) combined increasing cut-in speeds and feathering blades, with treatments in the first half and second half of nights at a wind energy facility in West Virginia resulted in fatality reductions of 50–72%; these results supported the hypothesis that bat activity and fatalities can sometimes be highest during the first half of the night. However, Hein et al. (2013) in another West Virginia study did not find significant differences in fatalities when the first and second half of the night were used as treatments. Good et al. (2013) conducted a series of cut-in speed experiments at a wind energy facility in western Indiana, which resulted in fatality reductions of 36–78%. Martin et al. (2017) combined near real-time information about wind speed and temperature at a wind energy facility in Vermont, which resulted in fatality reductions of 34–78%. Schirmacher et al. (2018) used wind speed information measured on a meteorological tower compared to wind speed measured on turbine nacelles to compare 10- and 20-min rolling average blanket curtailment, and found that wind speed measured on a meteorological tower combined with 20-min rolling average wind speed measurements resulted in the largest mean reduction in estimated fatalities. The TIMR approach reported here used 7.9 m/s as the threshold for considering curtailment if bats were detected; this is higher than the 5.0, 6.5, and 6.9 m/s blanket curtailment options often considered in conservation planning documents (U.S. Fish and Wildlife Service 2016). Thus, the TIMR approach would be expected to provide some protection to bats above even the blanket 6.9 m/s curtailment approach, especially when bats are expected to be flying in wind speeds ≥7.0 m/s, as is seen at the BSGF site (Sutter and Schumacher 2017). Additionally, the TIMR threshold for curtailment could be changed as more information becomes available, and could be changed mid-way through a deployment season. However, it is currently unclear how changes in wind-speed thresholds would influence fatality rates.

This study used four ReBAT acoustic monitoring systems distributed among the 88 turbines at the ~4,200 ha wind facility (one ReBAT system per ~1,050 ha). It is not yet clear what the optimal number and placement of ReBAT system is on wind turbines, but this configuration of ReBAT systems was successful in providing an effective prediction of risk at this wind facility, even for hoary bats, which appear to sometimes fly without using echolocation (Corcoran and Weller 2018). These results suggest that bats may be visiting wind turbines in spatial and/or temporal clusters such that when one bat's vocalizations triggers curtailment this protects other bats that may be visiting the wind facility at about the same time. Collecting acoustic information associated with other turbines in addition to the turbines at which ReBAT systems are deployed might help clarify the optimal number and placement of ReBAT systems at a given wind facility. Likewise, combining acoustic information with videography may also help clarify how bat behavior and risk is correlated in space and time around wind turbines and across species. During future deployments, and after considering the layout of turbines at the wind facility, it would be reasonable to begin with one ReBAT system per ~1,000 ha across the facility, then add or remove ReBAT systems as needed in future years, ideally within an adaptive management framework (Williams 2011). The acoustic data collected by these ReBAT systems can also provide daily acoustic post-construction monitoring for the site and would provide daily information about bat activity patterns at the facility. This information should allow biologists and managers to develop a significantly improved understanding of bat activity at the site, and how risk varies among species and is influenced by turbine location, season, environmental conditions, and time of night.

Several North American studies have reported estimates of the economic impacts of the treatments employed. Baerwald et al. (2009) reported a loss of \$3,000–\$4,000 (Canadian \$) for the 1-month study at 15 turbines. Arnett et al. (2011) reported an estimated loss in power generation of <1%/yr, and Martin et al. (2017) reported an estimated loss in power generation of ~1%/yr. The power generation and revenue loss resulting from the TIMR treatment reported here was estimated to be ≤3.2%/yr for the year of the study, which is higher than the loss reported by Arnett et al. (2011) and Martin et al. (2017). It is not entirely clear why the estimated loss observed at BSGF was higher, but it is possible that power and revenue generation could be improved substantially over this study by decreasing the upper wind speed threshold for curtailment (e.g., from 7.9 to 6.9 m/s, or lower). Given that theoretical power generation is proportional to the cube of wind speed (Gripe 2004), the curtailment threshold of 7.9 m/s used in this study would be expected to result in substantially less power generation and additional revenue loss when compared to a TIMR treatment using curtailment thresholds of, for example, 5.0, 6.5, or 6.9 m/s; this would, however, likely result in additional fatalities. The BSGF site also experiences relatively lower wind speeds on average when compared to many other wind energy facility locations in North America (Sutter and Schumacher 2017). The Vestas V82 turbines used at BSGF have power curves that increase rapidly with increasing wind speed

between the cut-in speed of 3.5 m/s and the peak production value of 13.5 m/s, and rapidly increase power generation between ~7.0 and 8.0 m/s. Therefore, it is likely that power and revenue loss due to TIMR smart curtailment at BSGF would typically be substantially <3%/yr on average and could be substantially improved with an upper curtailment threshold of <8.0 m/s. We estimate that curtailment time for treatment turbines was ~48.5% less than would have been expected under a blanket curtailment rule of 6.9 m/s, which should represent a substantial economic advantage for some wind energy producers.

The current study is the first that we are aware of in North America to explicitly incorporate information about the seasonal and diurnal wind speed regime along with details of overnight localized margin prices and tax credits available to wind producers. It is possible that the lower wind speed regime experienced at BSGF was responsible for some of the difference in economic impact at the BSGF site when compared to other study sites in North America. Reporting of information about local wind speed regimes, along with margin prices and tax credit calculations, would improve comparisons among study sites. Additionally, future analyses and research could focus on how to make decisions about the curtailment threshold that balance bat fatality reduction with maintaining economic performance at wind energy facilities.

Compared to North America, fewer experimental studies have tested the efficacy of operational mitigation in Europe (Beucher et al. 2011, Korner-Nievergelt et al. 2013). In one of the only published European studies of operational mitigation that we are aware of, which was conducted in southern France, the authors estimated that bat fatalities were reduced by 97–98% by raising the turbine cut-in speed to 6.5 m/s and turning off a light at the foot of the turbine towers (Beucher et al. 2011). However, despite the lack of experimental research, due to the conservation status of bat species in most European countries, operational mitigation is more common than it is in North America (Behr et al. 2017). Some European countries and states (such as some German federal states) require operational mitigation to reduce bat fatalities at wind energy facilities (Behr et al. 2017), and have strict fatality targets (e.g., ≤2 bats/turbine; Behr et al. 2017). Recent research in Germany has shown promising results in reducing bat fatalities using correlative statistical models (e.g., N-mixture models; Royle 2004) in combination with prior acoustic and environmental information to help guide near real-time curtailment decisions (Behr et al. 2017). However, without concurrent comparisons of approaches at the same or similar study sites, it is unclear how these approaches would compare to the TIMR smart curtailment approach presented here.

Bat ecologists and others have expressed concerns that the influences of wind energy development in North America can potentially have non-trivial population-level impacts on some bat populations of conservation concern (Cryan 2011, Hayes 2013, Smallwood 2013, Arnett et al. 2016, Frick et al. 2017). However, because of their nocturnal activity patterns, relatively small size, and tendency for reclusive behavior, it is extremely challenging to estimate population sizes and trends for bat species over large spatial scales, such as at ecoregional and continental scales (O'Shea et al. 2003, Hallam and Federico 2009, Hayes and Adams 2017). As a result, there is currently a lack of understanding of the continental-scale population dynamics of most North American bat species. Biologists are still learning about the basic ecology of some species of conservation concern in North America, and still do not clearly understand the ecological and genetic factors that shape and influence distributions, migration patterns, seasonal roost selection, and mating behavior of some North American species. As examples, despite being among the species most affected by wind energy development, bat ecologists in North America are only starting to realize that many silver-haired bats may spend the winter months in the interior west (Bonewell et al. 2017), and the first direct evidence of movements over long distances by an individual migratory bat was not published until recently (Weller et al. 2016). Furthermore, patterns of occurrence of bat species and individual bats at wind energy facilities and around wind turbines are the results of highly complex combinations of ecological, behavioral, and environmental processes, and these processes likely vary substantially among populations, species, species assemblages, ecoregions, and continents, and may also vary substantially from year to year. Thus, we unequivocally emphasize that the results presented here are seen as an initial step in understanding how operational mitigation and smart curtailment models can be successfully implemented to reduce bat fatalities at wind energy facilities in the various ecoregions of North America, while improving power production and adding operational flexibility relative to other approaches.

We also emphasize that the TIMR smart curtailment approach will need to be demonstrated as useful at reducing bat fatalities in other parts of the Midwestern United States and in other North American ecoregions (e.g., the northern and southern Great Plains, the Appalachian Mountains, the Northeastern United States, and in offshore environments in North America). We emphatically highlight that future smart curtailment studies should strive to overcome some of the potential weaknesses of this study. At the time this study was planned it was not yet commonly recognized that curtailment treatments can potentially influence the spatial patterns of how carcasses fall inside and beyond search plots. The study plan used here also did not measure vegetation cover and height inside search plots and link this information to detection probability estimates. We also emphasize that the ecological and environmental processes that influence the patterns of carcass deposition in space and time, carcass detectability once they

are available for detection, persistence of carcasses within plots, and the human dimensions processes that influence how searchers perceive and detect carcasses are all highly complex processes that would likely require years of research to fully understand at any given study site. As an example of the challenges inherent in understanding how ecological, environmental, and human dimensions variables can influence detectability associated with vertebrates, Lardner et al. (2015) and Rodda et al. (2015) analyzed the detectability of lizards associated with a multi-decade study site on Guam. Lardner et al. (2015) used >9,000 transect searches to evaluate the influence of 20 variables on detectability, including searcher identity, environmental covariates, and survey time, and used a multi-model inference approach to select highest ranking models (Burnham and Anderson 2002). This effort resulted in the best models only explaining a relatively small fraction of the variation in detectability. Rodda et al. (2015) also analyzed the detectability of lizards on Guam associated with the same study area and evaluated the stability of patterns in detectability over a 17-yr period. This research included removing all vegetation from some enclosed plots to compile total census data for these plots for the target species, and comparing this information to plot population estimates and detectability using standard techniques. These researchers concluded that even under the "auspicious" conditions of their study using 17 yr of data, which implicitly included the relatively abundant access to funding, well-qualified field crews, and continued participation and coordination among key personnel and stakeholders associated with such long-term ecological research programs: "The range of conditions under which population indices will provide useful data are. . .correspondingly small (perhaps even minute) and not yet well delimited" (Rodda et al. 2015:520). With the caveats of Rodda et al. clearly in mind, we view our study as but one brick in an emerging structure of knowledge (Forscher 1963) related to the ecological and environmental processes that influence bat fatalities at wind energy facilities and how smart curtailment approaches can help reduce these impacts on bat populations within the context of an adaptive management and continuous improvement framework (Williams 2011).

Given that fatality estimation protocols and models are continually improving, in future research it will be important to convincingly demonstrate that the carcass distributions in geographic space around wind turbines are not substantially different between control and treatment groups, and that if they are, these differences are incorporated into fatality estimates. As time and resources are available we also recommend: incorporating thorough carcass fall distribution studies; increasing the size, and perhaps changing the shape, of search plots to ensure that the carcass distributions associated with control and treatment turbines are clearly understood; including detectability covariates, such as searcher identity, environmental conditions including temperature and precipitation during and preceding surveys, time of surveys, size and species of carcasses, as well as fine-scale habitat conditions and structure, and evaluating their relative influence; conducting searches over the full activity period for bats in the area, such as from spring through autumn; using fatality estimation models that are capable of more fully incorporating the variability in these processes (e.g., the forthcoming generalized mortality estimator, GenEst; *available online*);[12] and conducting fatality estimation studies over multiple years, and when possible in multiple locations. It seems likely that it will require decades of continued research to more fully understand the influences of various curtailment approaches on bat populations in North America and on other continents.

Despite these caveats, it is possible that at many wind energy facilities smart curtailment using the TIMR approach may be sufficient to meet the current biological and economic goals of wind energy facility operators, wildlife agencies, and other stakeholders in a variety of ecoregions and operational contexts. By responding to current local bat activity and wind conditions in near real-time, the TIMR smart curtailment model can be viewed as having the potential to cut the Gordian knot of these complexities in some ecoregions. It would also be reasonable to hypothesize that this approach is likely to be successful in some other North American ecoregions and on other continents. Because this smart curtailment model uses just two parameters (current bat activity and current wind speed), the decision rule used can be adaptively modified for different locations, wind producer needs, and other constraints. For example, after the first year of deployment of a TIMR system at a given site, the bat activity and wind speed thresholds that trigger curtailment could be changed to reduce fatality levels, or to increase power production if fatalities are maintained below acceptable levels. Likewise the curtailment intervals could be changed as more information becomes available. For example, it is possible that reducing the curtailment time frame after the initial 30-min period from 10 min to 5 min might allow increased economic benefits while maintaining reasonable reductions in bat fatalities. Acoustic filters could also be conceivably added that would focus the risk model's attention on certain species of particular conservation concern. Other information, such as precipitation status and ambient temperature, could also be considered for inclusion to help tune site-specific models. The TIMR model has the added benefit of requiring little prior information about the timing or extent of bat activities at a given site prior to deployment and makes no statistical assumptions about links between bat activity, fatality risk, and environmental conditions. The TIMR approach could also be combined with information related to wind energy facility power purchase

[12] https://github.com/ddalthorp/GenEst

agreements, with seasonal habitat suitability estimates derived from species distribution and ecological niche models (Hayes et al. 2015), and/or in combination with predictive statistical models that predict fatality risk based on past bat behavior patterns and current environmental conditions (Behr et al. 2017).

On a continental scale, the current lack of reliable population estimates continues to make evaluating the impacts of wind energy on bats and related conservation and management planning challenging, especially in the face of potentially additive threats to North American bat populations, such as diseases (e.g., white-nose syndrome; Frick et al. 2010) and climate induced population declines (e.g., in western North America; Hayes and Adams 2017). Even under conditions that are optimal for population growth, temperate zone insectivorous bat species usually experience slow population growth rates due to the tendency of these species to give birth to one viable offspring per reproductive year and high mortality rates during a bat's first year of life (O'Shea et al. 2004). Given their contribution to mammalian species richness and diversity in North America and globally (Wilson and Reeder 2005), along with their ecological and economic importance (Ghanem and Voigt 2012), it will be helpful to continue monitoring and research related to operational mitigation and smart curtailment tools and on the current and future impacts of wind energy development on bat populations in North America and elsewhere. Efforts aimed at monitoring and estimating bat occupancy rates, distributions, and populations throughout North America (e.g., the North American Bat Monitoring Program; Loeb et al. 2015) are also likely to be useful in improving our understanding of the influences of wind energy development on continental-scale bat population trends.

Acknowledgments

This research was sponsored by the Electric Power Research Institute (EPRI). We sincerely acknowledge the support of the following organizations: We Energies, Duke Energy Corporation, Alliant Energy Corporation, and Exelon Corporation. Special recognition is given to Blue Sky Green Field Wind Energy Facility Manager, S. Schueller, and the Vestas support staff who assisted with the installation and maintenance of equipment. We are grateful to the insights and assistance provided by C. Sutter and C. Newman. J. Champion created the estimateN_custom function we used with the R package carcass. We thank S. Allen, T. Allison, E. Baerwald, S. Barnum, J. Carter, M. Costello, P. Cryan, G. Forcey, C. Hein, J. Rowan, M. Schirmacher, C. Thalken, J. Willmott, and two anonymous reviewers, whose feedback improved the content of this manuscript. M. Hayes dedicates his work on this project to J. Nuessel whose work in bat conservation over many years is helping to conserve sensitive populations in the West. ReBAT is a registered trademark and TIMR is a registered service mark of Normandeau Associates. TIMR is used with permission from Electric Power Research Institute.

Literature Cited

Alvarez, S. T., and W. Z. Lidicker Jr. 2015. Managing coexistence for bats and wind turbines. Therya 6:505–513.

American Wind Wildlife Institute [AWWI]. 2017. Wind turbine interactions with wildlife and their habitats: a summary of research results and priority questions. Washington, D.C., USA. www.awwi.org

Amorim, F., H. Rebelo, and L. Rodrigues. 2012. Factors influencing bat activity and mortality at a wind farm in the Mediterranean region. Acta Chiropterologica 14:439–457.

Arnett, E. B., and E. F. Baerwald. 2013. Impacts of wind energy development on bats: implications for conservation. Pages 435–456 *in* R. A. Adams and S. C. Pedersen, editors. Bat evolution, ecology, and conservation. Springer, New York, New York, USA.

Arnett, E. B., et al. 2008. Patterns of bat fatalities at wind energy facilities in North America. Journal of Wildlife Management 72:61–78.

Arnett, E. B., M. M. Huso, M. R. Schirmacher, and J. P. Hayes. 2011. Altering turbine speed reduces bat mortality at wind-energy facilities. Frontiers in Ecology and the Environment 9:209–214.

Arnett, E. B., C. Hein, M. R. Schirmacher, M. M. P. Huso, and J. M. Szewczak. 2013. Evaluating the effectiveness of an ultrasonic acoustic deterrent for reducing bat fatalities at wind turbines. PLoS ONE 9:e65794.

Arnett, E. B., E. F. Baerwald, F. Mathews, L. Rodrigues, A. Rodríguez-Durán, J. Rydell, R. Villegas-Patraca, and C. C. Voigt. 2016. Impacts of wind energy development on bats: a global perspective. Pages 295–323 *in* C. C. Voigt and T. Kingston, editors. Bats in the Anthropocene: conservation of bats in a changing world. Springer International Publishing, New York, New York, USA.

Aronson, J., E. Richardson, K. MacEwan, D. Jacobs, W. Marais, S. Aiken, P. Taylor, S. Sowler, and C. Hein. 2014. South African good practice guidelines for operational monitoring for bats at wind energy facilities. South African Bat Assessment Advisory Panel, Johannesburg, South Africa.

Baerwald, E. F., and R. M. R. Barclay. 2011. Patterns of activity and fatality of migratory bats at a wind energy facility in Alberta, Canada. Journal of Wildlife Management 75:1103–1114.

Baerwald, E. F., J. Edworthy, M. Holder, and R. M. Barclay. 2009. A large-scale mitigation experiment to reduce bat fatalities at wind energy facilities. Journal of Wildlife Management 73:1077–1081.

Banerjee, A., E. Prehoda, R. Sidortsov, and C. Schelly. 2017. Renewable, ethical? Assessing the energy justice potential of renewable electricity. AIMS Energy 5:768–797.

Behr, O. and von Helversen O. 2006. Gutachten zur Beeinträchtigung im freien Luftraum jagender und ziehender Fledermäuse durch bestehende Windkraftanlagen. Wirkungskontrolle zum Windpark "Roßkopf" (Freiburg i. Br.) im Jahr 2005. Unveröffentlichtes Gutachten im Auftrag der regiowind GmbH, Freiburg, Erlangen. [A study of the impairment of free air space foragers and migrating bats by existing wind turbines. Impact control of the wind farm "Roßkopf" (Freiburg i Br.) in 2005. Unpublished report on behalf of regiowind GmbH, Freiburg, Erlangen, Germany.]

Behr, O., R. Brinkmann, K. Hichradel, J. Mages, F. Korner-Nievergelt, I. Niermann, M. Reich, R. Simon, N. Weber, and M. Nagy. 2017. Mitigating bat mortality with turbine-specific curtailment algorithms: a model based approach. Pages 135–160 *in* J. Köppel, editor. Wind energy and wildlife interactions. Springer International, Cham, Germany.

Bernard, E., A. Paese, R. B. Machado, and L. M. de Souza Aguiar. 2014. Blown in the wind: bats and wind farms in Brazil. Natureza & Conservação 12:106–111.

Beucher, Y., V. Kelm, M. Geyelin and D. Pick. 2011. Parc éolien de Castelnae-Pégayrols (12): suivi évaluation post-implantation de l'impact sur les chauves souris. Bilan de campagne de la deuxième année d'exploitation (2009). Note synthétique extradite du rapport de Mars 2010. [Castelnae-Pégayrols wind farm (12): monitoring post-implementation assessment of the impact on bats; overview of the second year of operation (2009): summary of the March 2010 report], Ecosphere, Strasbourg, France.

Bonewell, L. R., M. A. Hayes, N. LaMantia-Olson, E. Wostl, and K. W. Navo. 2017. Silver-haired bats associated with abandoned mines in Colorado provide insights into winter habitat and roost use. Western North American Naturalist 77:404–407.

Brown, W., and B. Hamilton. Monitoring of bird and bat collisions with wind turbines at the Summerview Wind Power Project, Alberta 2005–2006. Terrestrial & Aquatic Environmental Managers, Calgary, Alberta, Canada.

Burnham, K. P., and D. R. Anderson. 2002. Model selection and multimodel inference: a practical information-theoretic approach, 2nd edition.. Springer, New York, New York, USA.

Chu, S., and A. Majumdar. 2012. Opportunities and challenges for a sustainable energy future. Nature 488(7411):294.

Clarke, B., E. Fokoué, and H. H. Zhang. 2009. Principles and theory for data mining and machine learning. Springer, New York, New York, USA.

Corcoran, A. J., and T. J. Weller. 2018. Inconspicuous echolocation in hoary bats (*Lasiurus cinereus*). Proceedings of the Royal Society B 285:20180441.

Crawley, M. J. 2013. The R book. Second edition. John Wiley & Sons, West Sussex, UK.

Cryan, P. M. 2003. Seasonal distribution of migratory tree bats (Lasiurus and Lasionycteris) in North America. Journal of Mammalogy 84:579–593.

Cryan, P. M. 2008. Mating behavior as a possible cause of bat fatalities at wind turbines. Journal of Wildlife Management 72:845–849.

Cryan, P. M. 2011. Wind turbines as landscape impediments to the migratory connectivity of bats. Environmental Law 41:355–370.

Cryan, P. M., and R. M. R. Barclay. 2009. Causes of bat fatalities at wind turbines: hypotheses and predictions. Journal of Mammalogy 90:1330–1340.

Cryan, P. M., et al. 2014*a*. Behavior of bats at wind turbines. Proceedings of the National Academy of Sciences USA 111:15126–15131.

Cryan, P. M., C. A. Stricker, and M. B. Wunder. 2014*b*. Continental-scale, seasonal movements of a heterothermic migratory tree bat. Ecological Applications 24:602–616.

Cryan, P. M., D. C. Dalton, and P. M. Gorresen. 2016. Selectively perceptible wind turbine system. United States Patent Application Publication: US 2016(0169501):A1.

DeCarolis, J. F., and D. W. Keith. 2006. The economics of large-scale wind power in a carbon constrained world. Energy Policy 34:395–410.

Doty, A. C., and A. P. Martin. 2013. Assessment of bat and avian mortality at a pilot wind turbine at Coega, Port Elizabeth, Eastern Cape, South Africa. New England Journal of Zoology 40:75–80.

Drewitt, A. L., and R. H. Langston. 2006. Assessing the impacts of wind farms on birds. Ibis 148(s1):29–42.

Erickson, W. P., J. Jeffrey, K. Kronner, and K. Bay. 2004. Stateline Wind Project Wildlife. Monitoring Final Report, July 2001–December 2003. Technical report peer-reviewed by and submitted to FPL Energy, the Oregon Energy Facility Siting Council, and the Stateline Technical Advisory Committee, Northwest Wildlife Consultants, Inc., Pendleton, Oregon, USA.

Forscher, B. K. 1963. Chaos in the brickyard. Science 142:339.

Frick, W. F., J. F. Pollock, A. C. Hicks, K. E. Langwig, D. S. Reynolds, G. G. Turner, C. M. Butchkoski, and T. H. Kunz. 2010. An emerging disease causes regional population collapse of a common North American bat species. Science 329:679–682.

Frick, W. F., E. F. Baerwald, J. F. Pollock, R. M. R. Barclay, J. A. Szymanski, T. J. Weller, A. L. Russell, S. C. Loeb, R. A. Medellin, and L. P. McGuire. 2017. Fatalities at wind turbines may threaten population viability of a migratory bat. Biological Conservation 209:172–177.

Gasparatos, A., C. N. Doll, M. Esteban, A. Ahmed, and T. A. Olang. 2017. Renewable energy and biodiversity: Implications for transitioning to a Green Economy. Renewable and Sustainable Energy Reviews 70:161–184.

Ghanem, S. J., and C. C. Voigt. 2012. Increasing awareness of ecosystem services provided by bats. Advances in the Study of Behavior 44:279–302.

Gibson, L., E. N. Wilman, and W. F. Laurance. 2017. How green is 'green' energy? Trends in Ecology and Evolution 32:922–935.

Good, R. E., M. Sonnenberg, and S. Simon. 2013. Bat evaluation monitoring studies at the Fowler Ridge Wind Farm, Benton County. Western EcoSystems Technology, Bloomington, Indiana, USA.

Gorresen, P. M., A. C. Miles, C. M. Todd, F. J. Bonaccorso, and T. J. Weller. 2008. Assessing bat detectability and occupancy with multiple automated echolocation detectors. Journal of Mammalogy 89:11–17.

Gorresen, P. M., P. M. Cryan, D. C. Dalton, S. Wolf, J. A. Johnson, C. M. Todd, and F. J. Bonaccorso. 2015. Dim ultraviolet light as a means of deterring activity by the Hawaiian hoary bat *Lasiurus cinereus cinereus*. Endangered Species Research 28:249–257.

Gripe, P. 2004. Wind power: renewable energy from home, farm, and business. Chelsea Green Publishing Company, White River Junction, Vermont, USA.

Gruver, J., K. Nasman and P. Rabie. 2016. Post-Construction Studies for the Blue Sky Green Field Wind Energy Facility, Fond du Lac County, Wisconsin. Final Fatality Report: June 2105 – October 2015. Prepared for S. Schumacher, We Energies, Milwaukee, Wisconsin. Prepared by Western EcoSystems Technology, Inc (WEST), Laramie, Wyoming, USA.

Hallam, T. G., and P. Federico. 2009. Applications of dynamic population models to bats. Pages 177–194 *in* T. H. Kunz and S. Parsons, editors. Ecological and behavioral methods for the study of bats. Second edition. The Johns Hopkins University Press, Baltimore, Maryland, USA.

Hanagasiogl, M., J. Aschwanden, F. Bontadina and M. D. l. Puente Nilsson. 2015. Investigating the effectiveness of bird and bat detection of the DTBat and DTBird systems at Calandawind turbine. Report to Swiss Federal Office of Energy and Swiss Federal Office of the Environment, Bern, Switzerland.

Hayes, M. A. 2013. Bats killed in large numbers at United States wind energy facilities. BioScience 63:975–979.

Hayes, M. A., and R. A. Adams. 2017. Simulated bat populations erode when exposed to climate change projections for western North America. PLoS ONE 12:e0180693.

Hayes, M. A., P. M. Cryan, and M. B. Wunder. 2015. Seasonally-dynamic presence-only species distribution models for a cryptic migratory bat impacted by wind energy development. PLoS ONE 10:e0132599.

Hein, C. D., and M. R. Schirmacher. 2016. Impact of wind energy on bats: a summary of our current knowledge. Human-Wildlife Interactions 10:19.

Hein, C. D., A. Prichard, T. Mabee and M. R. Schirmacher. 2013. Effectiveness of an operational mitigation experiment to reduce bat fatalities at the Pinnacle Wind Farm, Mineral County, West Virginia, 2012. An annual report submitted to Edison Mission Energy and the Bats and Wind Energy Cooperative. Bat Conservation International. Austin, Texas, USA.

Horn, J. W., E. B. Arnett, and T. H. Kunz. 2006. Behavioral responses of bats to operating wind turbines. Journal of Wildlife Management 72:123–132.

Hosmer, D. W., S. Lemeshow, and R. X. Sturdivant. 2013. Applied logistic regression, 3rd edition. Wiley, Hoboken, New Jersey, USA.

Hull, C. L., and L. Cawthen. 2013. Bat fatalities at two wind farms in Tasmania, Australia: bat characteristics, and spatial and temporal patterns. New Zealand Journal of Zoology 40:5–15.

Huso, M. 2011. An estimator of wildlife fatality from observed carcasses. Environmetrics 22:318–329.

Intergovernmental Panel on Climate Change [IPCC] 2014. Summary for policy makers. *In:* Climate Change 2014: Mitigation of Climate Change. Contribution of Working Group III to the Fifth Assessment Report of the Intergovernmental Panel on Climate Change. Cambridge University Press, Cambridge, UK.

Jacobson, M. Z., M. A. Delucchi, G. Bazouin, Z. A. Bauer, C. C. Heavey, E. Fisher, S. B. Morris, D. J. Piekutowski, T. A. Vencill, and T. W. Yeskoo. 2015. 100% clean and renewable wind, water, and sunlight (WWS) all-sector energy roadmaps for the 50 United States. Energy & Environmental Science 8:2093–2117.

Jameson, J. W., and C. K. R. Willis. 2014. Activity of tree bats at anthropogenic tall structures: implications for mortality of bats at wind turbines. Animal Behaviour 97:145–152.

Katzner, T., V. Bennett, T. Miller, A. Duerr, M. Braham, and A. Hale. 2016. Wind energy development: methods for assessing risks to birds and bats pre-construction. Human-Wildlife Interactions 10:42.

Kerns, J., W. P. Erickson, and E. B. Arnett. 2005. Bird and bat fatality at wind energy facilities in Pennsylvania and West Virginia. Pages 24–95 *in* E. B. Arnett, W. P. Erickson, J. Kerns, and J. Horn, editors. Relationships between bats and wind turbines in Pennsylvania and West Virginia: an assessment of fatality search protocols, patterns of fatality, and behavioral interactions with wind turbines. Bats and Wind Energy Cooperative, Bat Conservation International, Austin, Texas, USA.

Kiesecker, J. M., H. Copeland, A. Pocewicz, and B. McKenney. 2009. Development by design: blending landscape-level planning with the mitigation hierarchy. Frontiers in Ecology and the Environment 8:261–266.

Korner-Nievergelt, F., P. Korner-Nievergelt, O. Behr, I. Niermann, R. Brinkmann, and B. Hellriegel. 2011. A new method to determine bird and bat fatality at wind energy turbines from carcass searches. Wildlife Biology 17:350–363.

Korner-Nievergelt, F., R. Brinkmann, I. Niermann, and O. Behr. 2013. Estimating bat and bird mortality occurring at wind energy turbines from covariates and carcass searches using mixture models. PLoS ONE 8:e67997.

Korner-Nievergelt, F., I. Niermann, O. Behr, M. A. Etterson, R. Brinkmann, P. Korner, B. Hellriegel, T. Roth, M. M. P. Huso and D. Dalthorp. 2016. Package carcass: Estimation of the number of fatalities from carcass searches. https://cran.r-project.org/web/packages/carcass/index.html

Kunz, T. H., E. B. Arnett, W. P. Erickson, A. R. Hoar, G. D. Johnson, R. P. Larkin, M. D. Strickland, R. W. Thresher, and M. D. Tuttle. 2007. Ecological impacts of wind energy development on bats: Questions, hypotheses, and research needs. Frontiers in Ecology and Environment 5:315–324.

Kurta, A. 2017. Mammals of the Great Lakes region. Third edition. University of Michigan Press, Ann Arbor, Michigan, USA.

Kuvlesky Jr., W. P., L. A. Brennan, M. L. Morrison, K. K. Boydston, B. M. Ballard, and F. C. Bryant. 2007. Wind energy development and wildlife conservation: challenges and opportunities. Journal of Wildlife Management 71:2487–2498.

Lardner, B., G. Rodda, A. A. Yackel Adams, J. A. Savage, and R. N. Reed. 2015. Detection rates of geckos in visual surveys: turning confounding variables into useful knowledge. Journal of Herpetology 49:522–532.

Lehnert, L. S., S. Kramer-Schadt, S. Schönborn, O. Lindecke, I. Niermann, and C. C. Voigt. 2014. Wind farm facilities in Germany kill noctule bats from near and far. PLoS ONE 9: e103106.

Loeb, S. C., et al. 2015. A plan for the North American bat monitoring program (NABat). U.S. Department of Agriculture, Asheville, North Carolina, USA.

Lovich, J. E., and J. R. Ennen. 2013. Assessing the state of knowledge of utility-scale wind energy development and operation on non-volant terrestrial and marine wildlife. Applied Energy 103:52–60.

Marques, A. T., H. Batalha, S. Rodrigues, H. Costa, M. J. R. Pereira, C. Fonseca, M. Mascarenhas, and J. Bernardino. 2014. Understanding bird collisions at wind farms: an updated review on the causes and possible mitigation strategies. Biological Conservation 179:40–52.

Martin, C. M. 2015. Effectiveness of operational mitigation in reducing bat mortality and an assessment of bat and bird fatalities at the Sheffield Wind Facility, Vermont. Thesis. Department of Biological Sciences, Texas Tech University, Lubbock, Texas. The economic efficiency of operational mitigation. Journal of Mammalogy 98:378–385.

Martin, C. M., E. B. Arnett, R. D. Stevens, and M. C. Wallace. 2017. Reducing bat fatalities at wind energy facilities while improving economic efficiency of operational mitigation. Journal of Mammalogy 98:378–385.

Nicholls, B., and P. A. Racey. 2007. Bats avoid radar installations: could electromagnetic fields deter bats from colliding with wind turbines? PLoS ONE 2:e297.

Nicholls, B., and P. A. Racey. 2009. The aversive effect of electromagnetic radiation on foraging bats–a possible means of discouraging bats from approaching wind turbines. PLoS ONE 4:e6246.

Omernik, J. M., S. S. Chapman, R. A. Lillie, and R. T. Dumke. 2000. Ecoregions of Wisconsin. Transactions of the Wisconsin Academy of Sciences, Arts, and Letters 88:77–103.

O'Shea, T. J., M. A. Bogan, and L. E. Ellison. 2003. Monitoring trends in bat populations of the United States and territories: status of the science and recommendations for the future. Wildlife Society Bulletin 31:16–29.

O'Shea, T. J., L. E. Ellison, and T. R. Stanley. 2004. Survival estimation in bats: historical overview, critical appraisal, and suggestions for new approaches. Pages 297–336 *in* W. L. Thompson, editor. Sampling rare or elusive species: concepts, designs, and techniques for estimating population parameters. Island Press, Washington, D.C., USA.

Peste, F., et al. 2015. How to mitigate impacts of wind farms on bats? A review of potential; conservation measures in the European context. Environmental Impact Assessment Review 51:10–22.

Plyant, C. L., D. M. Nelson, M. C. Fitzpatrick, J. E. Gates, and S. R. Keller. 2016. Geographic origins and population

genetics of bats killed by wind-energy facilities. Ecological Applications 26:1381–1395.

R Core Team. 2018. R: A language and environment for statistical computing. R Foundation for Statistical Computing, Vienna, Austria. https://www.R-project.org

Rodda, G. H., K. Dean-Bradley, E. W. Campbell, T. H. Fritts, B. Lardner, A. A. Yackel Adams, and R. N. Reed. 2015. Stability of detectability over 17 years at a single site and other lizard detection comparisons from Guam. Journal of Herpetology 49:513–521.

Rodhouse, T. J., P. C. Ormsbee, K. M. Irvine, L. A. Vierling, J. M. Szewczak, and K. T. Vierling. 2015. Establishing conservation baselines with dynamic distribution models for bat populations facing imminent decline. Diversity and Distributions 21:1401–1413.

Rodrigues, L., et al. 2014. Guidelines for consideration of bats in wind farm projects, Revision 2014. EUROBATS Publication Series No. 6. ENEP/EUROBATS Secretariat, Bonn, Germany.

Rodríguez-Durán, A., and W. Feliciano-Robles. 2015. Impact of wind facilities on bats in the Neotropics. Acta Chiropterologica 17:365–370.

Roscioni, F., H. Rebelo, D. Russo, M. L. Carranza, M. Di Febbraro, and A. Loy. 2014. A modelling approach to infer the effects of wind farms on landscape connectivity for bats. Landscape Ecology 29:891–903.

Royle, J. A. 2004. N-mixture models for estimating population size from spatially replicated counts. Biometrics 60:108–115.

Rupprecht, C. 1980. Seasonal activity patterns in the bat community at Neda Mine. Field Stations Bulletin 13:1–7.

Rydell, J. L., M.-J. Bach, M. Dubourg-Savage, L. Rodrigues Green, and A. Hedenström. 2010. Bat mortality at wind turbines in northwestern Europe. Acta Chiropterologica 12:261–274.

Saidur, R., N. A. Rahim, M. R. Islam, and K. H. Solangi. 2011. Environmental impact of wind energy. Renewable and sustainable energy reviews 15:2423–2430.

Samuels, M. L., J. A. Witmer, and A. A. Schaffner. 2012. Statistics for the life sciences, 4th edition. Prentice Hall, New York, New York, USA.

Sánchez-Zapata, J. A., et al. 2016. Pages 97–123 *in* R. Mateo, B. Arroyo, and J. T. Garcia, editors. Effects of renewable energy production and infrastructure on wildlife. Current trends in wildlife research. Springer International Publishing, Cham, Switzerland.

Santos, H., L. Rodrigues, G. Jones, and H. Rebelo. 2013. Using species distribution modelling to predict bat fatality risk at wind farms. Biological Conservation 157:178–186.

Schirmacher, M. R., A. Prichard, T. Mabee, and C. Hein. 2018. Evaluating a novel approach to optimize operational minimization to reduce bat fatalities at the Pinnacle Wind Farm, Mineral County, West Virginia, 2015. An annual report submitted to NRG Energy and the Bats and Wind Energy Cooperative. Bat Conservation International, Austin, Texas, USA.

Smallwood, K. S. 2013. Comparing bird and bat fatality-rate estimates among North American wind-energy projects. Wildlife Society Bulletin 37:19–33.

Sutter, C., and S. Schumacher. 2017. Bat detection and shutdown system for utility-scale wind turbines. Technical Update 3002009038. Electric Power Research Institute, Palo Alto, California, USA.

Szewczak, J. M., and E. B. Arnett. 2007. Field test results of a potential acoustic deterrent to reduce bat mortality from wind turbines. Humboldt State University and Bat Conservation International, Austin, Texas, USA.

U.S. Fish and Wildlife Service [USFWS]. 2012. US Fish and Wildlife Service land-based wind energy guidelines. U.S. Fish and Wildlife Service. https://www.fws.gov/ecological-services/es-library/pdfs/WEG_final.pdf

U.S. Fish and Wildlife Service [USFWS]. 2016. Environmental Assessment for proposed Habitat Conservation Plan and Incidental Take Permit, Wildcat Wind Farm, Tipton and Madison Counties, Indiana. U.S. Fish and Wildlife Service, Bloomington Ecological Services Field Office, Bloomington, Indiana, USA.

Vestas. 2005. General specification, V82-1.65 MW MK II, NM82/1650 Version 2. Technical Document. www.edprnorthamerica.com

Voigt, C. C., A. G. Popa-Lisseanu, I. Niermann, and S. Kramer-Schadt. 2012. The catchment area of wind farms for European bats: a plea for international regulations. Biological Conservation 153:80–86.

Voigt, C. C., L. S. Lehnert, G. Petersons, F. Adorf, and L. Bach. 2015. Wildlife and renewable energy: German politics cross migratory bats. European Journal of Wildlife Research 61:213–219.

Voigt, C. C., O. Lindecke, S. Schönborn, S. Kramer-Schadt, and D. Lehmann. 2016. Habitat use of migratory bats killed during autumn at wind turbines. Ecological Applications 26:771–783.

Wagner, H.-J., and J. Mathur. 2013. Introduction to wind energy systems: basics, technology and operations. Second edition. Springer, Berlin, Germany.

Weller, T. J., and J. A. Baldwin. 2012. Using echolocation monitoring to model bat occupancy and inform mitigations at wind energy facilities. Journal of Wildlife Management 76:619–631.

Weller, T. J., K. T. Castle, F. Liechti, C. D. Hein, M. R. Schirmacher, and P. M. Cryan. 2016. First direct evidence of long-distance seasonal movements and hibernation in a migratory bat. Scientific Reports 6:34585.

Williams, B. K. 2011. Adaptive management of natural resources—framework and issues. Journal of Environmental Management 92:1346–1353.

Willmott, J. R., G. M. Forcey, and L. A. Hooton. 2015. Developing an automated risk management tool to minimize bird and bat mortality at wind facilities. Ambio 44: S557–S571.

Wilson, D. E., and D. M. Reeder, editors. 2005. Mammal species of the world: a taxonomic and geographic reference, 3rd edition. John Hopkins University Press, Baltimore, Maryland, USA.

Young Jr., D. P., S. Nomani, W. L. Tidhar, and K. Bay. 2011. NedPower Mount Storm wind energy facility post-construction avian and bat monitoring. Western EcoSystems Technology Inc, Cheyenne, Wyoming, USA.

Zerrahn, A. 2017. Wind power and externalities. Ecological Economics 141:245–260.

Zimmerling, J. R., and C. M. Francis. 2016. Bat mortality due to wind turbines in Canada. Journal of Wildlife Management 80:1360–1369.

Data Availability

Data are available on GitHub/Zenodo: https://doi.org/10.5281/zenodo.2566564
